# Targeting long noncoding RNA PMIF facilitates osteoprogenitor cells migrating to bone formation surface to promote bone formation during aging

**DOI:** 10.7150/thno.54477

**Published:** 2021-03-20

**Authors:** Dijie Li, Jin Liu, Chaofei Yang, Ye Tian, Chong Yin, Lifang Hu, Zhihao Chen, Fan Zhao, Ru Zhang, Aiping Lu, Ge Zhang, Airong Qian

**Affiliations:** 1 Lab for Bone Metabolism, Xi'an Key Laboratory of Special Medicine and Health Engineering, Key Lab for Space Biosciences and Biotechnology, School of Life Sciences, Northwestern Polytechnical University, Xi'an, China.; 2 Law Sau Fai Institute for Advancing Translational Medicine in Bone & Joint Diseases, School of Chinses Medicine, Hong Kong Baptist University, Hong Kong SAR, China.; 3 Shenzhen Institute for Research and Continuing Education (IRACE), Hong Kong Baptist University, Shenzhen, China.; 4 Research Center for Special Medicine and Health Systems Engineering, School of Life Sciences, Northwestern Polytechnical University, Xi'an, China.; 5 NPU-UAB Joint Laboratory for Bone Metabolism, School of Life Sciences, Northwestern Polytechnical University, Xi'an, China.

**Keywords:** long non-coding RNA, osteoprogenitor cells, cell migration, bone formation, aging

## Abstract

**Rationale:** The migration of mesenchymal osteoprogenitor cells (OPCs) to bone formation surface is the initial step of osteoblastogenesis before they undergo osteoblast differentiation and maturation for governing bone formation. However, whether the migration capacity of OPCs is compromised during aging and how it contributes to the aging-related bone formation reduction remain unexplored. In the present study, we identified a migration inhibitory factor (*i.e.*, long noncoding RNA PMIF) and examined whether targeting lnc-PMIF could facilitate osteoprogenitor cells migrating to bone formation surface to promote bone formation during aging.

**Methods:** Primary OPCs from young (6-momth-old) and aged (18-momth-old) C57BL/6 mice and stable lnc-PMIF knockdown/overexpression cell lines were used for *in vitro* and *in vivo* cell migration assay (*i.e.*, wound healing assay, transwell assay and cell intratibial injection assay). RNA pulldown-MS/WB and RIP-qPCR were performed to identify the RNA binding proteins (RBPs) of lnc-PMIF. Truncations of lnc-PMIF and the identified RBP were engaged to determine the interaction motif between them by RNA pulldown-WB and EMSA. By cell-based therapy approach and by pharmacological approach, small interfering RNA (siRNA)-mediated lnc-PMIF knockdown were used in aged mice. The cell migration ability was evaluated by transwell assay and cell intratibial injection assay. The bone formation was evaluated by microCT analysis and bone morphometry analysis.

**Results:** We reported that the decreased bone formation was accompanied by the reduced migration capacity of the bone marrow mesenchymal stem cells (BMSCs, the unique source of OPCs in bone marrow) in aged mice. We further identified that the long non-coding RNA PMIF (postulated migration inhibitory factor) (*i.e.*, lnc-PMIF) was highly expressed in BMSCs from aged mice and responsible for the reduced migration capacity of aged OPCs to bone formation surface. Mechanistically, we found that lnc-PMIF could bind to human antigen R (HuR) for interrupting the HuR-β-actin mRNA interaction, therefore inhibit the expression of β-actin for suppressing the migration of aged OPCs. We also authenticated a functionally conserved human lncRNA ortholog of the murine lnc-PMIF. By cell-based therapy approach, we demonstrated that replenishing the aged BMSCs with small interfering RNA (siRNA)-mediated lnc-PMIF knockdown could promote bone formation in aged mice. By pharmacological approach, we showed that targeted delivery of lnc-PMIF siRNA approaching the OPCs around the bone formation surface could also promote bone formation in aged mice.

**Conclusion:** Toward translational medicine, this study hints that targeting lnc-PMIF to facilitate aged OPCs migrating to bone formation surface could be a brand-new anabolic strategy for aging-related osteoporosis.

## Introduction

Bone is a dynamic organ that owns the potential to repair itself through the coordinated activity of osteoclasts derived from hemopoietic stem/progenitor cells (HSPCs) and osteoblasts arose from mesenchymal stem/progenitor cells (MSPCs) 1, 2. However, the self-repairing capacity of bone is markedly compromised during aging primarily due to the decreased osteoblastic bone formation, which could result in the porotic and fragile bone with increased risk of fracture as well as impaired fracture healing in the elder population 3, 4. The decreased bone formation during aging is previously considered to be partly attributed to the aging-related impairment of osteoblastogenesis from the mesenchymal osteoprogenitor cells (OPCs), *e.g.*, bone marrow-derived mesenchymal stem cells (BMSCs) and adipose-derived mesenchymal stem cells (AdMSCs), including the decreased cell proliferative capacity and decreased osteogenic differentiation potential with increased adipogenic tendency 4, 5. Intriguingly, a recent study demonstrated that aging did not alter the proliferative and osteogenic differentiation capacity of human AdMSCs *in vitro* 6, which is in line with a previous study showing the comparable bone forming capacity *in vivo* between the human BMSCs from young and elder donors after implantation in immune-deficient mice 7. These contradictory findings suggest that there could be other factors influencing the osteoblastogenesis of OPCs during aging.

The migration of OPCs to bone formation surface is the initial step of osteoblastogenesis before they undergo osteoblast differentiation and maturation for governing bone formation 8, 9. However, there is little knowledge about whether the migration capacity of OPCs is compromised during aging and how it could contribute to the aging-related bone formation reduction. Microtubule actin crosslinking factor 1 (MACF1), a spectraplakin protein that interacts with the cytoskeleton, plays an important role in cell migration 10-13. We previously found that Macf1 was significantly downregulated in aged osteoporotic patients 14. In the current study, we identified a long non-coding RNA PMIF (postulated migration inhibitory factor, *i.e.*, lnc-PMIF) which is transcribed from the Macf1 gene locus as another transcript. We showed that the decreased bone formation was accompanied by the upregulated expression of lnc-PMIF within BMSCs and the reduced migration capacity of BMSCs in aged mice. We found that the lnc-PMIF could bind to human antigen R (HuR) for interrupting the HuR-β-actin mRNA interaction, therefore inhibit the expression of β-actin for suppressing the migration of aged OPCs to bone formation surface. We also authenticated a functionally conserved human lncRNA ortholog of murine lnc-PMIF. We further showed that silencing lnc-PMIF could facilitate the aged OPC migrating to bone formation surface for promoting bone formation in aged mice.

## Results

### Decreased bone formation accompanied by reduced migration of OPCs to bone formation surface and elevated lnc-PMIF expression in OPCs during aging

**Figure 1:**
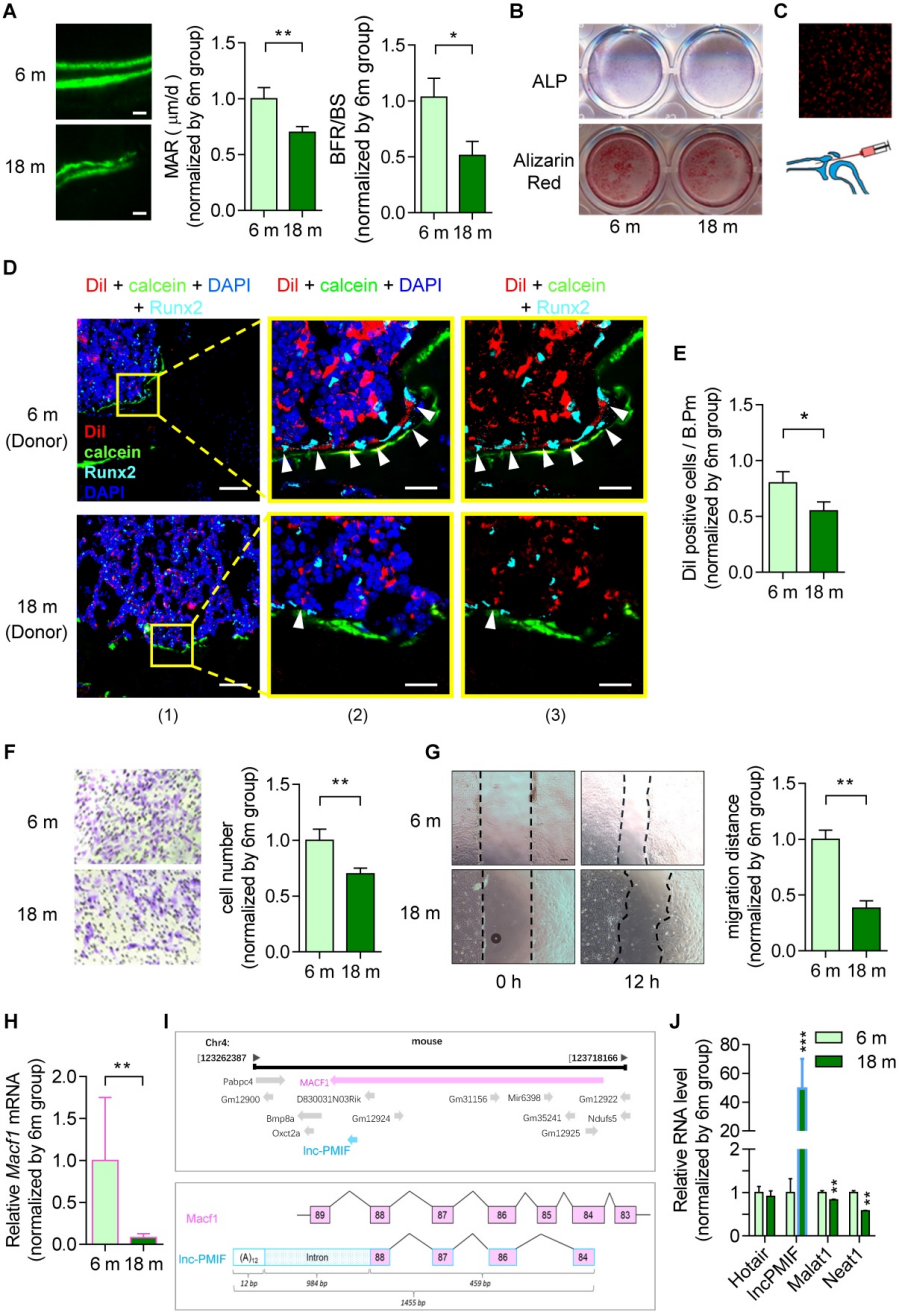
** Decreased bone formation accompanied by reduced migration of OPCs to bone formation surface and elevated lnc-PMIF expression in OPCs in male mice during aging. (A)** Dynamic bone histomorphometry of distal femoral metaphysis of young (6-month-old, 6 m) and aged (18-month-old, 18 m) mice. Top panel: the representative fluorescent images of new bone formation revealed by double calcein labeling. Bottom panel: the dynamic bone histomorphometric parameters (MAR and BFR/BS). (B) Representative images of ALP staining in BMSCs after 7 days of osteogenic induction (top) and Alizarin Red S staining in BMSCs after 14 days of osteogenic induction (bottom). Scale bar: 100 µm. (C) Schematic diagram for intratibial injection of Dil-labeled BMSCs. **(D)** Representative confocal images of tibia metaphysis showing the Dil-labeled cells (red) and Runx2-expressing cells (light blue) on and around the calcein-labeled bone formation surface (green) at 3 days after injection. Cell nucleus were stained by DAPI (dark blue). Scale bar: 100 µm (left panel) and 25 µm (middle and right panels). Arrow heads indicate the Dil-positive cells at the bone formation surface. **(E)** The average number of Dil-labeled cell approaching bone formation surface. n=3~4 mice per group. **(F)** Transwell migration assay of BMSCs *in vitro*. Left: Representative images of the migrated cells. Right: the number of migrated cells. **(G)** Wound healing assay of BMSCs *in vitro*. Left: Representative images of the miagrated cells. Right: the migration distance. **(H)** QPCR analysis of the expression of Macf1 in BMSCs isolated from the young (6 m) and aged mice (18 m), respectively. **(I)** Lnc-PMIF is a sense-overlapping transcript to the Macf1 gene. Schematic representation of the Macf1 and lnc-PMIF gene loci on mouse chromosome 4 (top). Exon composition of the lnc-PMIF transcript and organization of the overlapping exon with Macf1 (bottom). **(J)** QPCR analysis of the expression of Hotair, lncPMIF, Malat1 and Neat1 in BMSCs isolated from the 6 m and 18 m mice, respectively. *Note:* BMSCs were isolated from the young and aged mice, respectively. For* in vitro* assay the experiments were conducted in triplicates. For *in vivo* assay, n=6 mice per group unless specifically annotated. All data were expressed as mean ± SD. *P < 0.05, **P < 0.01 by Student's *t*-test.

To investigate the age-related change in bone formation, we collected bone specimens from the established aging murine model. Dynamic bone histomorphometry analysis showed that the mineralizing apposition rate (MAR) and bone formation rate (BFR/BS) were both significant lower in aged (18-month-old) C57BL/6 mice as compared to the young (6-month-old) control mice **(Figure 1A**, **Figure S1A)**, suggesting that the decreased bone formation in mice during aging. To investigate the age-related change in osteogenic potential of OPCs *in vitro*, we isolated the BMSCs, the unique source of OPCs in bone marrow, from the aforementioned mice, which were analyzed by RNA sequencing (RNA-Seq) and further induced for osteogenic differentiation *in vitro*. It was found that a series of gene encoding the migration-related proteins, *e.g*. the MMP family (*Mmp*-3, -9, -11, -13, -14), *CD44*, *β-actin*, *Tgf-β* and *Pdgf-BB*, were all downregulated in aged BMSCs as compared to young BMSCs in both genders (**Figure S2A**, **Figure S2B**). As revealed by the Alkaline phosphatase (ALP) staining and Alizarin Red S staining analysis, the ALP activity after 7 days of osteogenic induction and calcium mineral deposition after 14 days of osteogenic induction were comparable between the BMSCs from aged and young mice **(Figure 1B**, **Figure S1B)**, respectively, indicating that the osteogenic potential of BMSCs* in vtiro* was not altered during aging. Thereafter, we isolated the BMSCs again from the aged and young mice, which were proliferated *in vitro* followed by the labeling with Dil (a lipophilic membrane stain) and intratibial injection into another batch of young mice (1×10^6^ cells per mouse) **(Figure 1C)**. The tibias were then harvested three days later for undecalcified cryosection followed by fluorescent immunostaining of the osteogenic marker Runx2. As revealed by Immunofluorescent analysis, we surprisingly found that the average number of Dil-labeled cells approaching the calcein-labelled bone formation surface was significantly lower in mice treated by aged BMSCs as compared to those treated by young BMSCs, suggesting that the migration capacity of OPCs to the bone formation surface was reduced during aging **(Figure 1D-E**, **Figure S1C-D)**. Interestingly, the expression of Runx2 were detected in most of the Dil-labeled cells migrated to the calcein-labelled bone formation surface, indicating that the OPCs migrated to the bone formation surface could undergo osteogenic differentiation to contribute to bone formation *in vivo*. Consistently, as revealed by the Transwell migration assay and Wound healing assay, the migration capacity *in vitro* was remarkably compromised in aged BMSCs as compared to young BMSCs **(Figure 1F-G**, **Figure S1E-F)**.

It was found that the* Macf1* was significantly downregulated in aged BMSCs as compared to young BMSCs **(Figure 1H)**. Interestingly, we identified a long non-coding RNA PMIF (postulated migration inhibitory factor, *i.e.*, lnc-PMIF), which is transcribed from the Macf1 gene locus as another transcript **(Figure 1I)**. Subsequently, we performed QPCR analysis to compare the expression of lnc-PMIF and another three lncRNAs previously reported to be involved in regulating cell migration,* i.e.*, Hotair 15, Malat1 16 and Neat1 17, between the aged and young BMSCs. To our surprise, only the expression of lnc-PMIF were remarkably higher in aged BMSCs as compared to young BMSCs **(Figure 1J**, **Figure S1G)**. Collectively, all the above findings suggested that the decreased bone formation was accompanied by the reduced migration of OPCs to bone formation surface and the elevated lnc-PMIF expression in OPCs in mice during aging.

### Lnc-PMIF suppresses the migration of OPCs to bone formation surfaces

Next, we employed the MC3T3-E1 cell line, which is a common murine osteogenic progenitor cell line, to investigate the role of lnc-PMIF in OPCs. First, we obtained the full-length of lnc-PMIF by 3' Rapid Amplification of cDNA Ends (3' RACE) technique and confirm the sequences containing 1455 bp by Sanger sequencing **(Figure S3A**, **Additional Data File 1)**. We then examined the subcellular localization of lnc-PMIF by QPCR and fluorescence *in situ* hybridization (FISH) analysis, by which we found that the lnc-PMIF transcript was detected in both nuclei and cytoplasm of MC3T3-E1 cells** (Figure S3B-C)**. To determine the expression level of lnc-PMIF in osteoblast differentiation, MC3T3-E1 cells were cultured in osteogenic medium for 35 days. It was found that lnc-PMIF increased in the early stage and then decreased after mineralization initiation during osteoblast differentiation (**Figure S3D**), suggesting that lnc-PMIF might play a role in the early stage of osteogenic differentiation. To investigate the effect of lnc-PMIF on Macf1, we conducted cell transfection assay by using expression plasmids and siRNA of lnc-PMIF, respectively. It was found that overexpression of lnc-PMIF had no significant effect on Macf1 expression (**Figure S4A-B**). Importantly, knockdown of lnc-PMIF could upregulate the Macf1 expression in both RNA level and protein level (**Figure S4C-D**). In addition, neither overexpression nor knockdown of lnc-PMIF had notable effect on the coding and noncoding transcripts that transcribed from/nearby the lnc-PMIF locus (**Figure S4E-F**). To conduct the lnc-PMIF gain- and loss-of-function study, we transfected the MC3T3-E1 cells with the lnc-PMIF shRNA-expressing plasmid or nonsense RNA control (NC) shRNA-expressing plasmid to generate the osteogenic progenitor cell line with stable lnc-PMIF knockdown (KD) and the relative control (KD-NC), respectively **(Figure S5A)**. We also transfected the MC3T3-E1 cells with lnc-PMIF-expressing plasmid or NC-expressing plasmid to generate the osteogenic progenitor cell line with stable lnc-PMIF overexpression (OE) and the relative control (OE-NC), respectively **(Figure S5B)**. Interestingly, we found that the cell numbers, cell viability and the proportions of cell cycle phases were all comparable between the KD and KD-NC group and between the OE and OE-NC group, respectively **(Figure S5C-H)**. Moreover, there were no significant differences in either the mRNA and protein expression of osteoblast-specific markers (RUNX2, ALP and Collagen type I), ALP activity or calcium mineral deposition between the KD and KD-NC groups as well as between the OE and OE-NC groups after osteogenic induction **(Figure S5I-N)**. These findings indicated that lnc-PMIF was not involved in regulating the proliferation and osteogenic differentiation of OPCs.

**Figure 2:**
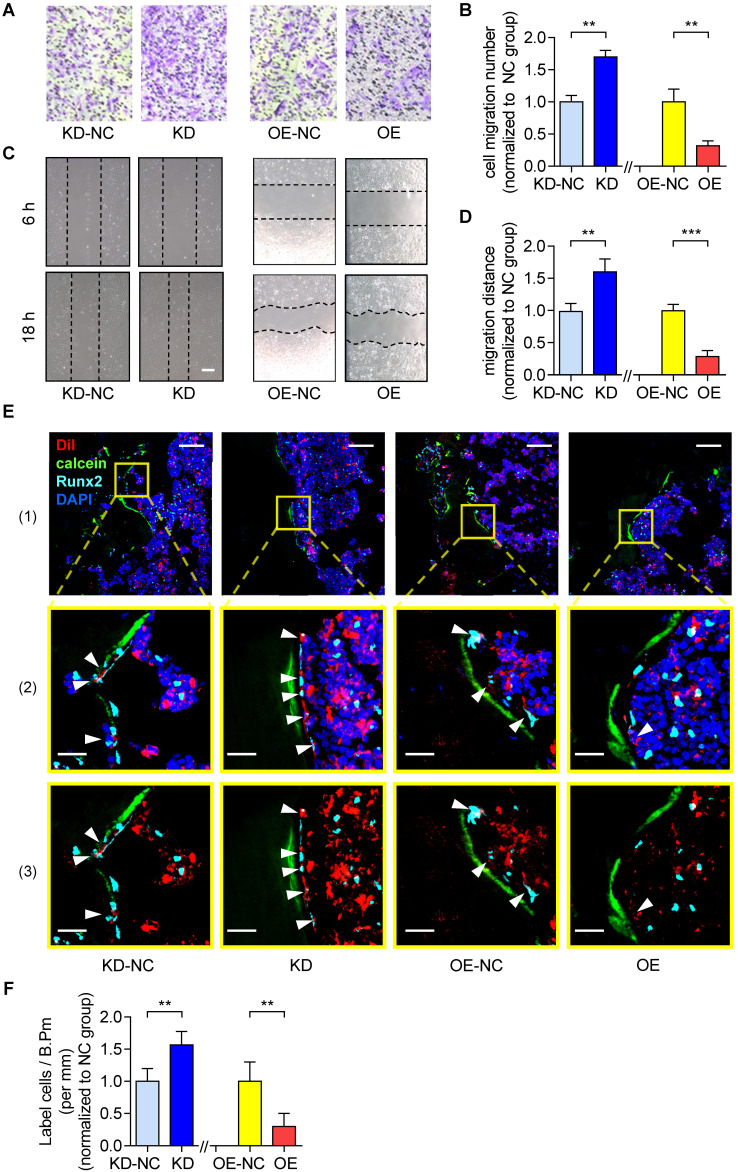
** Lnc-PMIF suppresses the migration of OPCs to bone formation surfaces**. **(A)** Representative images of the migrated cells by Transwell migration assay on MC3T3-E1 cells in different groups *in vitro*. **(B)** The number of migrated cells by by Transwell migration assay on MC3T3-E1 cells in different groups *in vitro*. **(C)** Representative images of the migrated cells by Wound healing assay on MC3T3-E1 cells in different groups *in vitro*. **(D)** The migration distance by Wound healing assay on MC3T3-E1 cells in different groups *in vitro*. **(E)** Representative confocal images of tibia metaphysis showing the Dil-labeled cells (red) and Runx2-expressing cells (light blue) on and around the calcein-labeled bone formation surface (green) at 3 days after injection. Cell nucleus were stained by DAPI (dark blue). Scale bar: 100 µm (top panel) and 25 µm (middle and bottom panels). Arrow heads indicate the Dil-positive cells at the bone formation surface.** (F)** The average number of Dil-labeled cell approaching bone formation surface. Note: KD: MC3T3-E1 cells with stable lnc-PMIF knockdown, KD-NC: MC3T3-E1 cells with stable nonsense control RNA transfection, OE: MC3T3-E1 cells with stable lnc-PMIF overexpression, OE-NC: MC3T3-E1 cells with stable nonsense control RNA overexpression. For* in vitro* assay the experiments were conducted in triplicates. For *in vivo* assay, n=3~4 mice per group. All data were expressed as mean ± SD. **P < 0.01, ***P < 0.001 by Student's *t*-test.

On the other hand, as revealed by Transwell migration assay and Wound healing assay, the cell migration number and the migration distance were both significantly higher in the KD group as compared to the KD-NC group **(Figure 2A-B)**. In contrast, they were both significantly lower in OE group as compared to the OE-NC group **(Figure 2C-D)**. Thereafter, we harvested the KD, KD-NC, OE and OE-NC cells, which were labelled by Dil and injected into the bone marrow cavity of tibias in young C57BL/6 mice (1×10^6^ cells per mouse) as afore described. The tibias were harvested three days later for the subsequent immunofluorescent analysis as aforementioned. We found that the average number of Dil-labeled cells approaching the calcein-labelled bone formation surface was significantly higher in the mice treated by KD cells as compared to those treated by KD-NC cells, whereas it was significantly lower in the mice treated by OE cells as compared to those treated by OE-NC cells **(Figure 2E-F)**. We then performed immunohistochemical (IHC) staining and found that the lnc-PMIF-related signaling molecules were higher in the mice treated by KD cells as compared to those treated by KD-NC cells **(Figure S6)**. Taken together, these findings suggested that lnc-PMIF could suppress the migration of OPCs to bone formation surfaces without disturbing their proliferation and osteogenic potential.

### Lnc-PMIF interacts with HuR to inhibit β-actin expression for suppressing OPC migration

**Figure 3:**
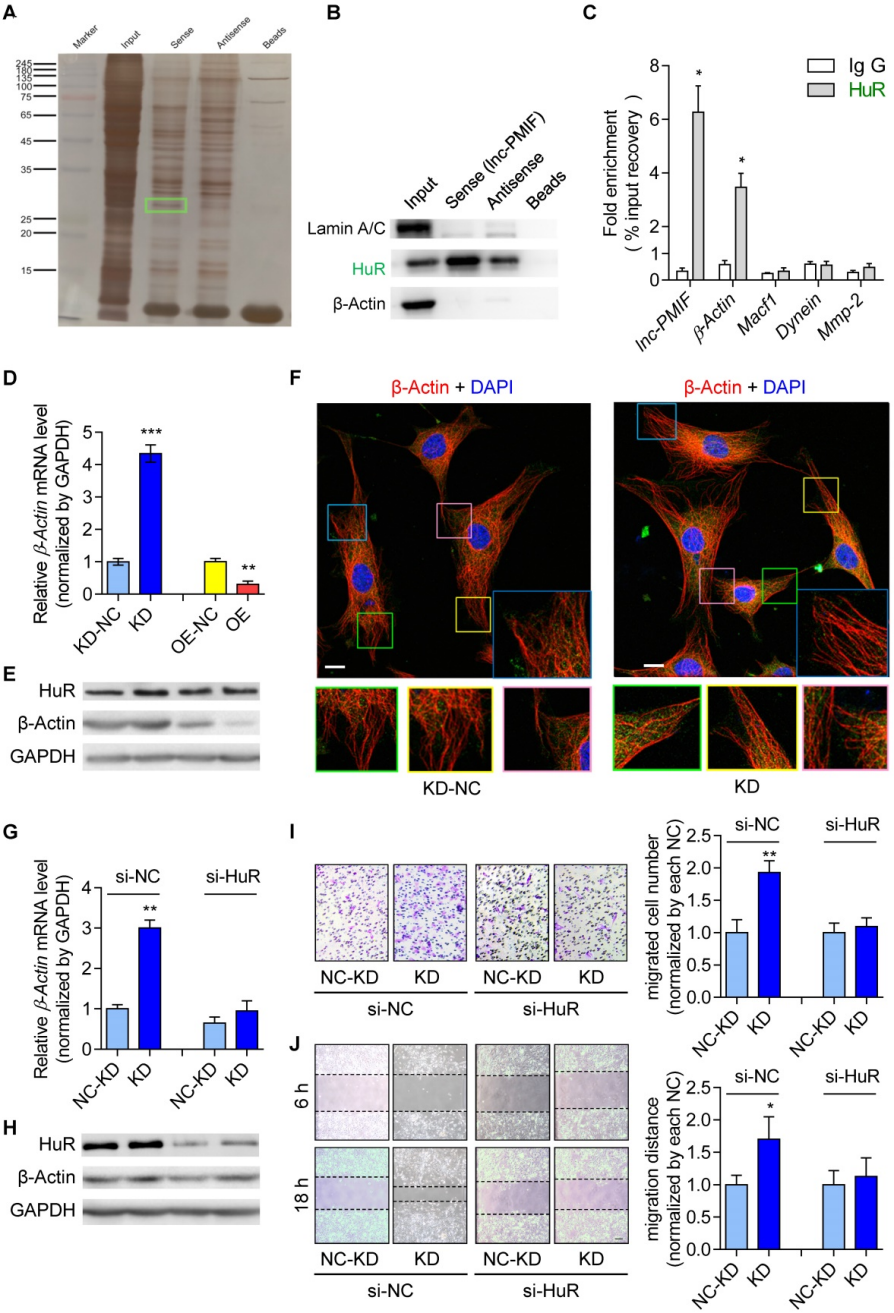
** Lnc-PMIF interacts with HuR to inhibit β-actin expression for suppressing OPC migration* in vitro*. (A)** The image of SDS-PAGE with silver staining for visualizing the pull-down fractions with biotin-labeled lnc-PMIF (sense) or biotin-labeled control RNA (antisense). The green box indicates the band area cut for the subsequent Mass Spectrometry for identifying the protein partners candidates of lnc-PMIF.** (B)** Western blot analysis of the detection of HuR, LaminA/C (as the protein control from nucleus) and β-actin (as the protein control from cytoplasm) in the pull-down fractions with biotin-labeled lnc-PMIF (sense) or biotin-labeled control RNA (antisense).** (C)** QPCR analysis of the detection of lnc-PMIF and the mRNA of β-actin, Macf1, Dynein and MMP-2 bound to the immunoprecipitated HuR proteins or IgG control proteins.** (D)** QPCR analysis of the β-actin mRNA expression in KD, KD-NC, OE and OE-NC cells, respectively. **(E)** Western blot analysis of the HuR, β-actin and GAPDH protein expression in KD, KD-NC, OE and OE-NC cells, respectively. **(F)** Representative confocal images of the β-actin fluorescent immunostaining in KD and KD-NC cells, respectively. The cellular area of the box in different colors were magnified. Scale bar: 10 µm. **(G)** QPCR analysis of the β-actin mRNA expression in KD and KD-NC cells transfected with HuR siRNA or NC-siRNA, respectively. **(H)** Western blot analysis of the HuR, β-actin and GAPDH protein expression in KD and KD-NC cells transfected with HuR siRNA or NC-siRNA, respectively.** (I)** Transwell migration assay on KD and KD-NC cells transfected with HuR siRNA or NC-siRNA, respectively. Left: representative images of the migrated cells. Right: the number of migrated cells. Note: KD: MC3T3-E1 cells with stable lnc-PMIF knockdown, KD-NC: MC3T3-E1 cells with stable nonsense control RNA transfection, OE: MC3T3-E1 cells with stable lnc-PMIF overexpression, OE-NC: MC3T3-E1 cells with stable nonsense control RNA overexpression. **(J)** Wound healing assay on KD and KD-NC cells transfected with HuR siRNA or NC-siRNA, respectively. Left: representative images. Right: quantification analysis of migration distance. All *in vitro* experiments were conducted in triplicates. All data were expressed as mean ± SD. ns: not statistically significant, *P < 0.05, **P < 0.01, ***P < 0.001 by Student's *t*-test.

Thereafter, we sought to understand the lnc-PMIF-mediated regulatory mechanism on OPC migration. First, we search for the interacting protein partners of lnc-PMIF by conducting RNA pulldown assay on the cell lysis of MC3T3-E1 cells transfected with biotin-labeled lnc-PMIF-expressing plasmid. The eluted pull-down fractions were resolved and separated by SDS-PAGE and visualized by silver staining, which showed that several proteins (with molecular weight around 45 kDa and between 25~35 kDa, respectively) were specifically pull-downed together with the biotin-labeled lnc-PMIF from the cell lysis of biotin-labeled lnc-PMIF-expressing cells as compared to the control cells **(Figure 3A)**. Subsequently, the bands containing the aforementioned pull-down fractions were cut and subjected for Mass Spectrometry (MS), by which we identified that the human antigen R (HuR), an RNA-binding protein previously found to promote cell migration and invasion 18, 19, was among the candidates for the interacting protein partners of lnc-PMIF **(Table S3)**. We then performed western blot analysis to confirm that HuR were enriched within the aforementioned pull-down fractions from the cell lysis of biotin-labeled lnc-PMIF-expressing cells **(Figure 3B)**. In turn, we conducted the RNA immunoprecipitation (RIP) assay in combination with QPCR analysis in the MC3T3-E1 cells, by which we detected a significantly higher level of lnc-PMIF bound to the immunoprecipitated HuR proteins as compared to that bound to the immunoprecipitated IgG control proteins **(Figure 3C)**. Interestingly, we also detected a significantly higher mRNA level of *β-actin*, one of the essential components of cell migration 20, bound to the immunoprecipitated HuR proteins as compared to that bound to the immunoprecipitated IgG control proteins **(Figure 3C)**, whereas the mRNA levels of the other three migration-related genes, *Macf1*, *Dynein* and *Mmp2*, bound to the immunoprecipitated HuR and IgG proteins were alike **(Figure 3C)**. All these findings confirmed that lnc-PMIF could directly bind to HuR.

Because HuR could bind to and stabilize the β-actin mRNA for promoting the migration and invasion of tumor cells 18, 19, we next investigated whether the HuR-β-actin-mediated regulatory mechanism were also involved in the regulation of OPC migration. Consistently, we found that the mRNA and protein expression of β-actin as well as the cell migration ability were all remarkably increased in MC3T3-E1 cells with HuR gene overexpression, while they were all markedly decreased in MC3T3-E1 cells with RNAi-mediated HuR gene knockdown **(Figure S7)**. Accordingly, we then investigate whether lnc-PMIF is involved in the HuR-β-actin-mediated regulation on OPC migration. We found that both the mRNA and protein expression of *β-actin* were significantly downregulated in the aforementioned MC3T3-E1 cells with stable lnc-PMIF gene overexpression (OE), but significantly upregulated in the aforementioned MC3T3-E1 cells with stable lnc-PMIF gene knockdown (KD) **(Figure 3D-E)**, indicating that lnc-PMIF could inhibit the *β-actin* expression in OPCs. Interestingly, we found that neither gene overexpression nor knockdown of lnc-PMIF could alter the protein expression of HuR **(Figure 3E)**. In addition, the confocal laser scanning microscopy of the β-actin fluorescent immunostaining showed that the OPC microfilaments (MF), which are composed of polymers of actin and have revealed their pivotal role in cell migration 21-23, were better arranged in the aforementioned KD cells as compared to the KD-NC cells **(Figure 3F)**. These findings, in combination with the aforementioned findings of Transwell migration and Wound healing assay of the KD, KD-NC, OE and OE-NC cells **(Figure 2A-D)**, suggested that lnc-PMIF could inhibit the expression of β-actin to suppress OPC migration without affecting the expression of HuR. To investigate whether HuR could mediate the regulation of lnc-PMIF on β-actin and OPC migration, we performed RNAi-mediated HuR gene knockdown in the aforementioned KD cells and KD-NC control cells, respectively. Interestingly, we found that the elevated mRNA and protein expression of β-actin in KD cells were remarkably downregulated when the HuR gene was simultaneously knockdown **(Figure 3G-H)**. Consistently, the increased cell migration ability of KD cells was also compromised when the HuR gene was simultaneously knockdown **(Figure 3I-J)**. Collectively, all these data suggested that lnc-PMIF could interact with HuR for inhibiting the expression of β-actin to suppress OPC migration. Meanwhile, we also identified a human lnc-PMIF ortholog **(Figure S6A**, **Additional Data File 2)**, which was transcribed from the human Macf1 gene locus as another transcript (**Figure S8A-C**). In line with the function of murine lnc-PMIF, we found that silencing of the human lnc-PMIF could directly inhibit the migration activity of the human osteoblastic precursor hFOB1.19 cells* in vitro* **(Figure S8D-E)**, indicating that the lnc-PMIF-mediated regulatory mechanism on OPC migration were functionally conserved between murine and human.

### lnc-PMIF bind to the RRM3 of HuR for interrupting the HuR-β-actin mRNA interaction to inhibit β-actin expression for suppressing OPC migration

**Figure 4:**
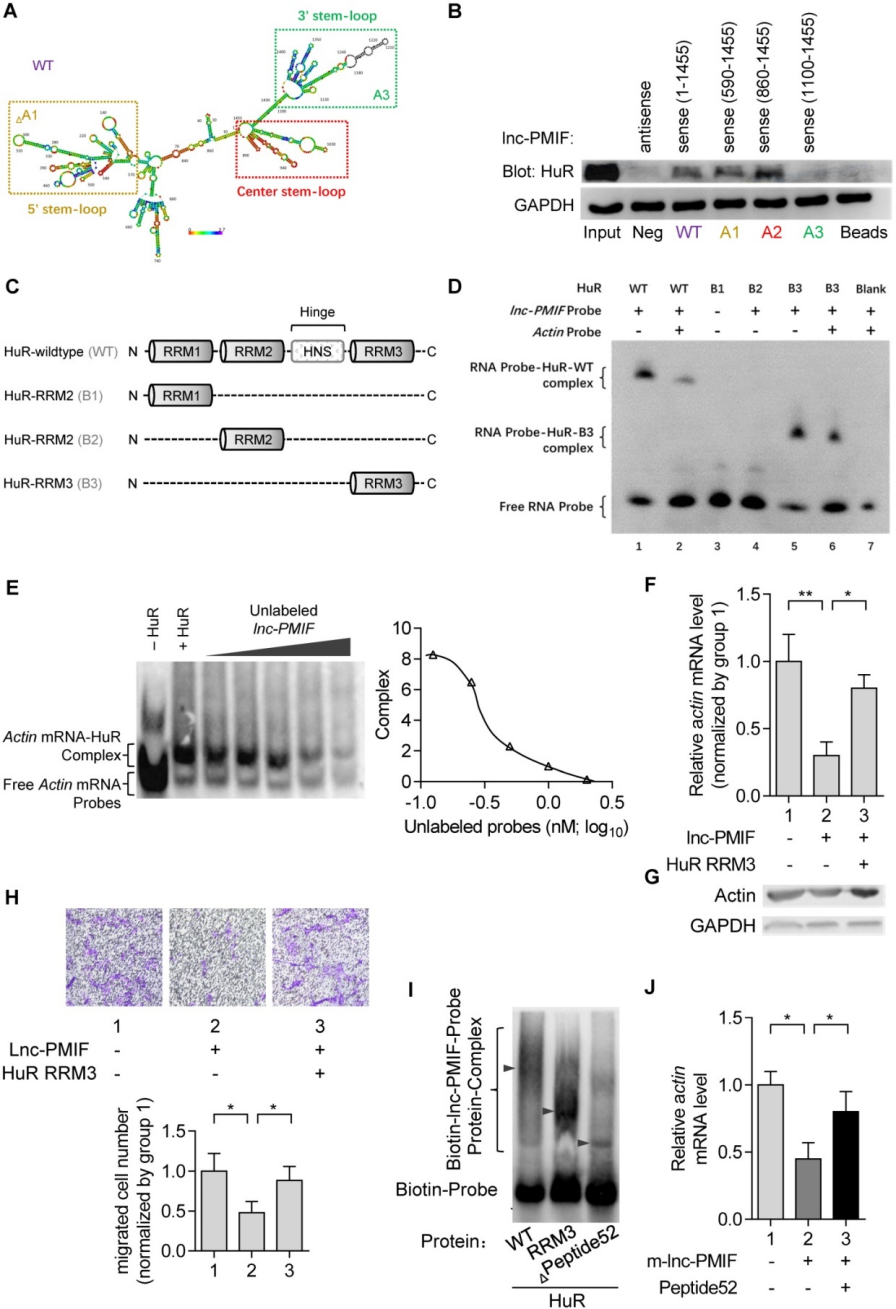
** lnc-PMIF bind to the RRM3 of HuR for interrupting the HuR-β-actin interaction to inhibit β-actin expression for suppressing OPC migration. (A)** The predicted secondary structure of lnc-PMIF. The red color indicates strong confidence for the prediction of each base. **(B)** RNA pull-down detection of the interaction between HuR and lnc-PMIF truncations. After the RNA pull-down experiment from MC3T3 cells by using antisense RNA, WT lnc-PMIF (sense, 1-1455 bp) and three truncated lnc-PMIFs (sense, 590-1455 bp, 860-1455 bp and 1100-1455 bp), respectively. HuR was detected by western blotting. **(C)** Schematic diagram showing the full length of WT HuR proteins and three truncated mutants (RRM1, RRM2 and RRM3). **(D)** RNA electrophoretic mobility shift assay (EMSA) of the interaction between lnc-PMIF and HuR truncations. The biotin-labeled lnc-PMIF probe was incubated with either WT HuR or truncated mutant B3 with or without unlabeled β-actin probe, respectively. The lnc-PMIF-HuR complex was detected by EMSA. **(E)** RNA EMSA of the competitive binding of HuR between β-actin mRNA and lnc-PMIF. The biotin-labeled β-actin probe was incubated with WT HuR in the absent or presence of various concentration of unlabeled lnc-PMIF probe, respectively. Left: The β-actin mRNA-HuR complex was detected by EMSA. Right: The quantitative curve of the complex detected. **(F)** QPCR analysis of the β-actin mRNA expression in OE-NC cells and OE cells with or without the overexpression of HuR RRM3, respectively. **(G)** Western blot analysis of the β-actin protein expression in OE-NC cells and OE cells with or without the overexpression of HuR RRM3, respectively. **(H)** Transwell migration assay on OE-NC cells and OE cells with or without the overexpression of HuR RRM3, respectively. Top: representative images of the migrated cells. Bottom: the number of migrated cells. **(I)** RNA-EMSA analysis of the interaction between lnc-PMIF and HuR-wildtype (WT) / HuR-RRM3 (RRM3) / HuR-RRM3-truncation (Leu251-II302) (Peptide52). **(J)** qPCR detection of actin after HuR-RRM3-Peptide52 transfected in lnc-PMIF overexpression MC3T3-E1 cells. ***Note:***OE: MC3T3-E1 cells with stable lnc-PMIF overexpression, OE-NC: MC3T3-E1 cells with stable nonsense control RNA overexpression. All *in vitro* experiments were conducted in triplicates. All data were expressed as mean ± SD. ns: not statistically significant, *P < 0.05, **P < 0.01 by Student's *t*-test.

To elucidate how lnc-PMIF interact with HuR to regulate β-actin expression and OPC migration, we first predicted the secondary structure of lnc-PMIF by bioinformatic analysis **(Figure 4A)**, and synthesized the biotinylated full-length lnc-PMIF (WT, sense 1-1455 bp) and the truncated lnc-PMIF mutants, *i.e.* mutant A1 (sense 590-1455 bp without the 5' stem-loop), mutant A2 (sense 860-1455 bp with the center stem-loop and 3' stem-loop) and mutant A3 (sense 1100-1455 bp with the 3' stem-loop alone), respectively. We then transfected the MC3T3-E1 cells with the aforementioned WT or mutant sequences and conducted tagged-RNA streptavidin pull-down assay. The subsequent western blot analysis showed that HuR was present in the pull-down fractions from the cells transfected with either WT lnc-PMIF, truncated lnc-PMIF mutant A1 or truncated lnc-PMIF mutant A2, but absent in the pull-down fractions from the cells transfected with truncated lnc-PMIF mutant A3 **(Figure 4B)**. These data indicated that the center stem-loop of lnc-PMIF were sufficient for enabling the interaction between lnc-PMIF and HuR.

Next, we synthesized the wildtype HuR proteins (WT) and the truncated mutants of HuR proteins (i.e., mutant B1 with the RNA recognition motif (RRM) 1 of HuR alone, mutant B2 with HuR RRM2 alone and mutant B3 with HuR RRM3 alone, respectively) **(Figure 4C)**. Meanwhile, we designed and synthesized the biotin-labeled or unlabeled probe specifically targeting the center-stem loop of lnc-PMIF and the biotin-labeled or unlabeled probe specifically targeting the sequences of β-actin mRNA previously reported for interacting with HuR protein, respectively. By RNA electrophoretic mobility shift assay (EMSA), we found that the lnc-PMIF-HuR complex could only be detected when the biotin-labeled lnc-PMIF probe was incubated with either WT HuR or truncated mutant B3 **(Figure 4D)**, suggesting that the RRM3 of HuR could mediate the interaction between HuR and lnc-PMIF. Intriguingly, the amount of lnc-PMIF -HuR complex detected was markedly reduced when the biotin-labeled lnc-PMIF probe was incubated either with WT HuR and unlabeled β-actin probe or with truncated mutant B3 and β-actin probe **(Figure 4D)**. Moreover, the β-actin mRNA-HuR complex could be detected when the biotin-labeled β-actin probe was incubated with WT HuR, whereas their amount detected was decreased after the concurrently addition of unlabeled lnc-PMIF probe in a dose-dependent manner **(Figure 4E)**. These data suggest that the lnc-PMIF could compete with β-actin mRNA to interact with HuR. Furthermore, we constructed the HuR-RRM3 expressing plasmid, which was transfected into the aforementioned OE MC3T3-E1 cells with stable lnc-PMIF gene overexpression. We found that the decreased mRNA and protein expression of β-actin in OE cells were remarkably upregulated after the overexpression of HuR-RRM3 **(Figure 4F-G)**. Consistently, the reduced cell migration activity in OE cells was notably enhanced after the overexpression of HuR-RRM3 **(Figure 4H)**. Further, we identified a cross-species functionally conserved HuR sequence (Peptide52, a 52 animo acids (aa) peptide on HuR-RRM3) binding lnc-PMIF **(Additional Data File 3**, **Figure 4I)**. Exogenous Peptide52 supplementation in cells could reverse the reduced actin level **(Figure 4J)** and promote cell migration **(Figure S9)**. Collectively, all these findings suggested that the lnc-PMIF could bind to the RRM3 of HuR for interrupting the HuR-β-actin interaction, by which it could inhibit β-actin expression for suppressing OPC migration.

### Silencing lnc-PMIF facilitates aged OPCs migrating to bone formation surface for promoting bone formation in aged mice

**Figure 5:**
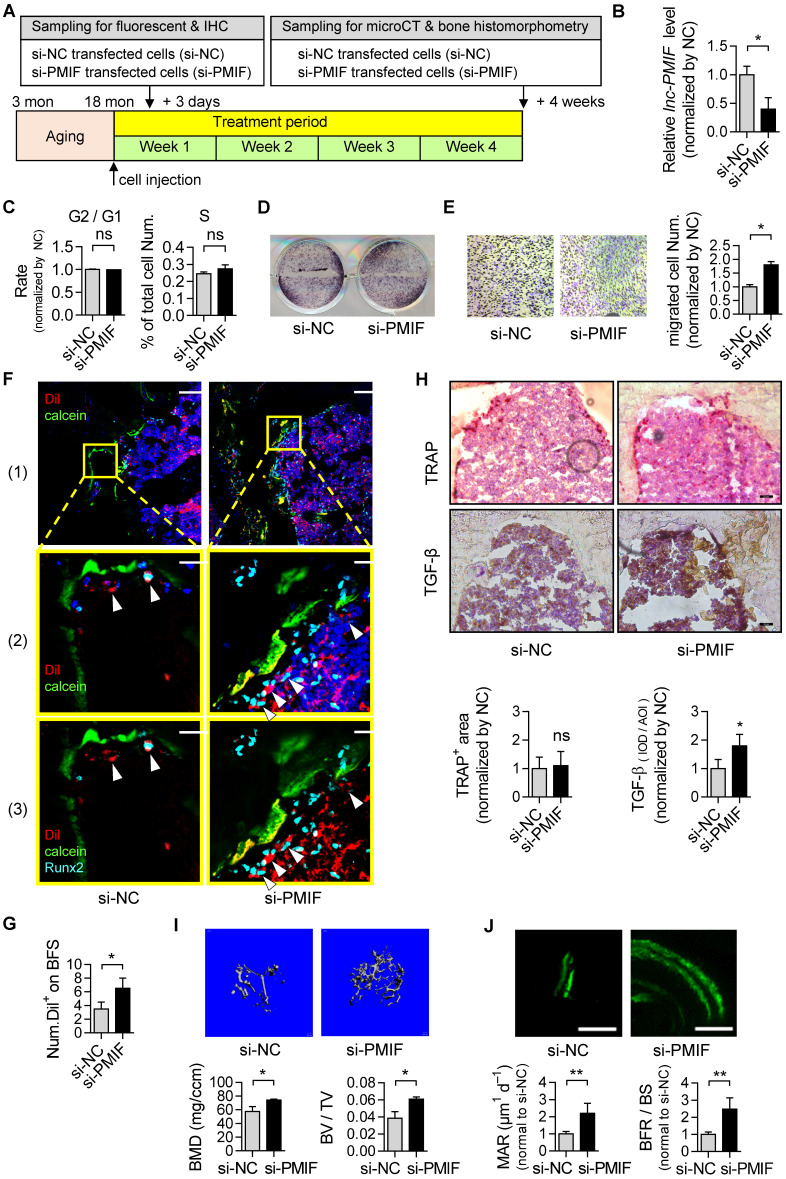
** Silencing of lnc-PMIF in aged OPCs facilitates them migrating to bone formation surface for promoting bone formation in aged mice. (A)** Schematic diagram of the experimental design. The aged BMSCs were harvested from aged mice and then transfected with lnc-PMIF siRNA (si-PMIF) or NC siRNA (si-NC), labeled with Dil and intratibially injected to the aged mice. **(B)** QPCR analysis of the lnc-PMIF expression in the aged BMSCs from different groups* in vitro*. **(C)** Flow cytometry analysis of the ratio of cells at G2 phase to the cells at G1 phase and the percentage of cells at S phase in the aged BMSCs from different groups during *in vitro* proliferation. **(D)** Representative images of ALP staining in in the aged BMSCs from different groups after 7 days of osteogenic induction* in vitro*. Scale bar: 100 µm. **(E)** Transwell migration assay on the aged BMSCs from different groups *in vitro*. Left: representative images of the migrated cells. Right: the number of migrated cells. **(F)** Representative confocal images of tibia metaphysis showing the Dil-labeled cells (red) and Runx2-expressing cells (light blue) on and around the calcein-labeled bone formation surface (green) in the mice from different groups at 3 days after injection. Cell nucleus were stained by DAPI (dark blue). Scale bar: 100 µm (top and middle) and 25 µm (bottom). Arrow heads indicate the Dil-positive cells at the bone formation surface. **(G)** The average number of Dil-labeled cell approaching bone formation surface. n=3~5 mice per group. **(H)** Immunohistochemical (IHC) staining of TRAP (top) and TGF-β (middle) at tibia metaphysis in the mice from different groups at 3 days after injection. Top, Middle: representative images, Bottom: quantification. Scale bar: 50 µm. **(I)** Micro-CT analysis of the proximal tibia metaphysis from the mice in different groups. Top panel: the representative micro-CT images showing the 3-D trabecular microarchitecture of proximal tibia metaphysis. Bottom panel: the micro-CT parameters (BMD and BV/TV). **(J)** Dynamic bone histomorphometry of the proximal tibia metaphysis from the mice in different groups. Top panel: the representative fluorescent images of new bone formation revealed by double calcein labeling. Bottom panel: the dynamic bone histomorphometric parameters (MAR and BFR/BS). *Note:* For* in vitro* assay, the experiments were conducted in triplicates. For *in vivo* assay, n=6 mice per group unless specifically annotated. All data were expressed as mean ± SD. *P < 0.05, **P < 0.01 by Student's *t*-test.

Considering the compromised migration capacity of aged OPCs to bone formation surface and the aberrantly upregulated lnc-PMIF in aged OPCs as aforementioned, we next sought to investigate whether silencing lnc-PMIF could facilitate the aged OPCs migrating to bone formation surface. The aged BMSCs were harvested and proliferated *in vitro* as aforementioned and transfected with lnc-PMIF small interference RNA (si-PMIF) for silencing lnc-PMIF and NC siRNA (si-NC) as control, respectively **(Figure 5A)**. QPCR analysis showed that the expression of lnc-PMIF was markedly downregulated in the si-PMIF-treated BMSCs as compared to the si-NC-treated BMSCs **(Figure 5B)**, which suggested the efficient knockdown of lnc-PMIF in the aged OPCs. Flow cytometry analysis showed no significant difference in the proportions of cell cycle phases between the si-PMIF-treated aged BMSCs and si-NC-treated aged BMSCs, indicating that knockdown of lnc-PMIF did not affect the proliferation of aged BMCSs **(Figure 5C)**. ALP activity staining revealed no significant differences in ALP activity between the si-PMIF-treated aged BMSCs and si-NC-treated aged BMSCs after osteogenic induction, suggesting that knockdown of lnc-PMIF did not alter the osteogenic capacity of aged OPCs *in vitro* **(Figure 5D)**. However, Transwell migration assay showed that the si-PMIF-treated aged BMSCs had a significantly higher cell migration number than that of si-NC-treated aged BMSCs **(Figure 5E)**. These data suggested that silencing lnc-PMIF could enhance the migration capacity of aged OPCs without disturbing the proliferation and osteogenic differentiation of aged OPCs *in vitro*. Thereafter, we performed intratibial injection with the Dil-labelled si-PMIF-treated aged BMSCs or Dil-labelled si-NC-treated aged BMSCs on the aged male C57BL/6 mice (1×10^6^ cells per mouse), which were sacrificed three days later to collect the tibias for the subsequent Immunofluorescent analysis as aforementioned **(Figure 5A)**. We found that the average number of Dil+ cells approaching the calcein-labelled bone formation surface was significantly higher in the mice receiving si-PMIF-treated aged BMSCs as compared to the mice receiving si-NC-treated aged BMSCs **(Figure 5F-G)**, suggesting that silencing lnc-PMIF could facilitate the aged OPCs migrating to bone formation surface in aged mice* in vivo*. More importantly, Runx2 expression was detected in most of the migrated Dil+ cells in the mice receiving the si-PMIF-treated aged BMSCs, indicating that the si-PMIF-treated aged BMSCs migrated to the bone formation surface could undergo normal osteogenic differentiation, which would contribute to bone formation in the aged mice. In addition, the average integral optical density (IOD) of TGF-β positive cell on bone surface was significantly higher, while TRAP positive area had no significant difference, in the mice receiving si-PMIF-treated aged BMSCs as compared to the mice receiving si-NC-treated aged BMSCs (**Figure 5H**), indicating that si-PMIF-treated aged BMSCs might affect cell recruitment (for bone formation) rather than bone resorption. To evaluate the effect of replenishing the aged BMSCs with lnc-PMIF knockdown as the cell-based therapy on aging-related osteoporosis, we performed intratibial injection with the aforementioned si-PMIF-treated aged BMSCs or si-NC-treated aged BMSCs on another batch of aged male C57BL/6 mice (1×10^6^ cells per mouse) **(Figure 5A)**. As shown by microCT analysis, we found the higher bone mass and superior microarchitecture of tibia metaphysis together with the higher BMD and BV/TV in the mice receiving si-PMIF-treated aged BMSCs as compared to the mice receiving si-NC-treated aged BMSCs on 4 weeks after the BMSC injection **(Figure 5H)**. Additionally, as revealed by dynamic bone histomorphometry analysis, we found the higher MAR and BFR/BS in the mice receiving si-PMIF-treated aged BMSCs as compared to the mice receiving si-NC-treated aged BMSCs on 4 weeks after the BMSC injection **(Figure 5I)**. These results demonstrated that replenishing the aged BMSCs with lnc-PMIF knockdown could promote bone formation in aged mice. Collectively, it also suggested that the aberrantly upregulated lnc-PMIF could be responsible for the reduced migration of aged OPCs to the bone formation surface, which could contribute to the aging-related bone formation reduction.

### Targeted delivery of lnc-PMIF siRNA approaching OPCs around bone formation surface promotes bone formation in aged mice

**Figure 6:**
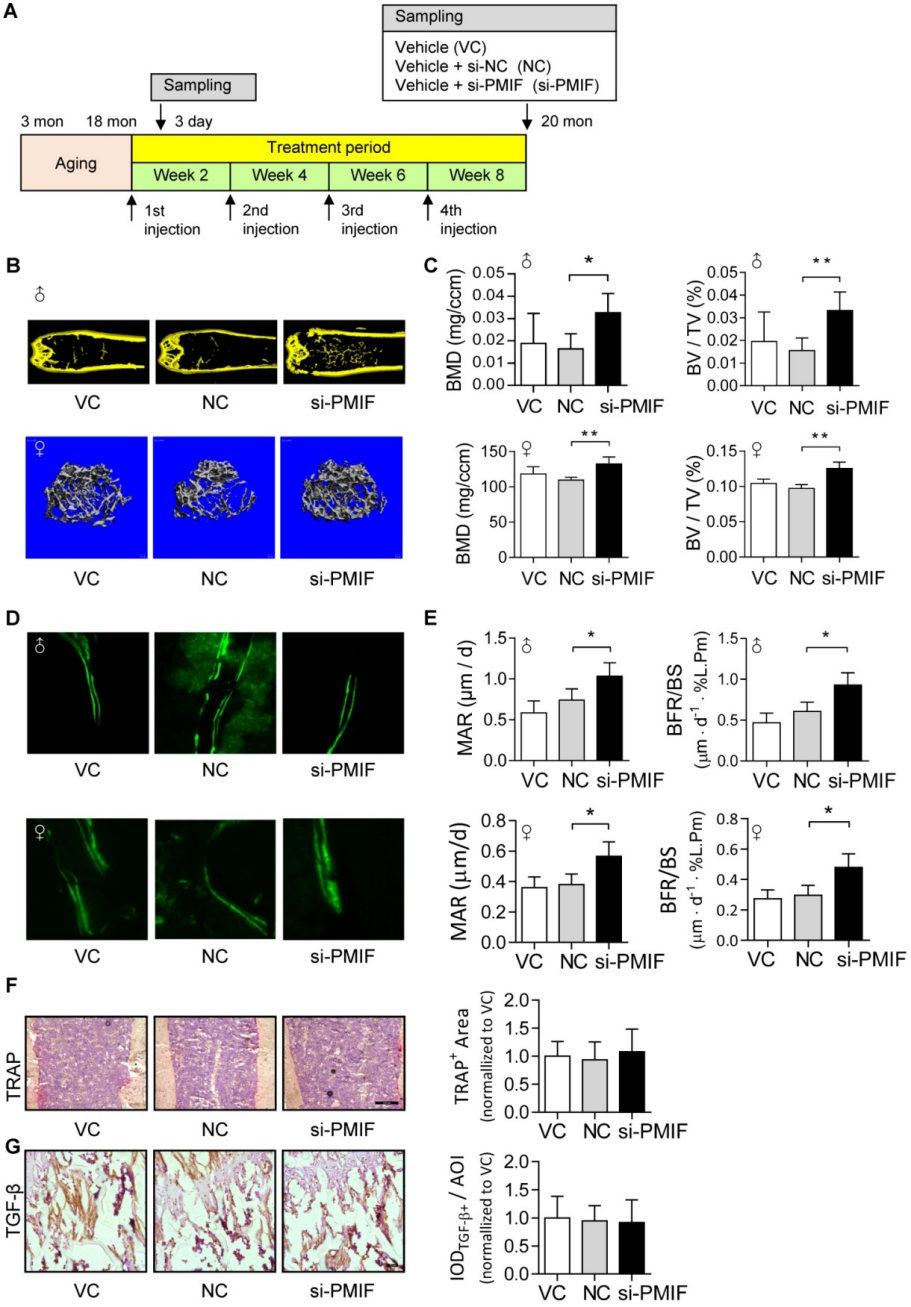
** Targeted delivery of lnc-PMIF siRNA approaching OPCs around bone formation surface promotes bone formation in aged mice. (A)** Schematic diagram of the experimental design. The aged male mice were treated with lnc-PMIF siRNA (si-PMIF) or NC siRNA (si-NC) encapsulated with (DSS_6_)-liposome, or (DSS_6_)-liposome alone (Vehicle) at a 2 weeks interval. **(B)** Representative micro-CT images showing the 3-D trabecular microarchitecture of the distal femur metaphysis from the mice with both genders in different groups. Upper panel: male mice. Lower panel: female mice. **(C)** The micro-CT parameters (BMD and BV/TV) in different groups. Upper panel: male mice. Lower panel: female mice. **(D)** Representative fluorescent images of calcein double labeling of the distal femur metaphysis from the mice with both genders in different groups. Upper panel: male mice. Lower panel: female mice. **(E)** The dynamic bone histomorphometric parameters (MAR and BFR/BS) in different groups. Upper panel: male mice. Lower panel: female mice. **(F)** TRAP staining at distal femur metaphysis from the above mice. Left: representative images. Right: quantification of TRAP-positive area. Scale bar: 100 µm. **(G)** IHC staining of TGF-β at distal femur metaphysis from the above mice. Left: representative images. Right: quantification of TGF-β positive area. Scale bar: 50 µm. *Note:* n=6 mice per group. All data were expressed as mean ± SD. *P < 0.05, **P < 0.01 by One-way ANOVA analysis followed by *post-hoc* test.

Given that the aging-related osteoporosis is a systemic bone disease, we next sought to evaluate the effect of systemically targeted silencing of lnc-PMIF in aged OPCs around the bone formation surface by pharmacological approach on aging-related osteoporosis. To facilitate the lnc-PMIF siRNA (*i.e.*, si-PMIF) approaching the OPCs around the bone formation surface, the si-PMIF or si-NC was encapsulated within our previously developed bone formation surface-targeting delivery system (*i.e.*, (DSS_6_)-liposome) which could facilitate the encapsulated siRNA approaching the low crystallized bone formation surface where OPCs are recruited for osteogenic differentiation 24. A batch of male and female C57BL/6 mice with natural aging at 21 months of age were intravenously injected with 4 periodic doses of (DSS_6_)-liposome-si-PMIF (10 mg/kg), (DSS_6_)-liposome-si-NC or (DSS_6_)-liposome alone (vehicle) at a 2 weeks interval **(Figure 6A)**. To examine the knockdown efficiency of si-PMIF, the Gli1+ mesenchymal OPCs previously shown to be responsible for cancellous bone formation 25 were either isolated from the cancellous bone region by fluorescent activated cell sorting (FACS) or collected around the calcein labeled bone formation surface by laser capture microdissection (LCM) in the mice of both genders from each group at 1 week after the first dose of treatment. QPCR analysis showed that the expression of lnc-PMIF was significantly lower in the Gli1+ cells in the si-PMIF-treated group as compared to the si-NC-treated and vehicle-treated groups **(Figure S10)**, suggesting the efficient gene knockdown of lnc-PMIF within OPCs around bone formation surface. At 23 months of age, all the mice were sacrificed to collect bone specimens for subsequent microCT and dynamic bone histomorphometry. As shown by microCT analysis, we found the higher bone mass and superior microarchitecture of tibia metaphysis together with the higher BMD and BV/TV in the mice with both genders from si-PMIF-treated group as compared to the si-NC-treated and vehicle-treated groups **(Figure 6B-C)**. As revealed by dynamic bone histomorphometry analysis, we found the higher MAR and BFR/BS in the mice with both genders from si-PMIF-treated group as compared to the si-NC-treated and vehicle-treated groups **(Figure 6D-E)**. In addition, after the treatment, neither the TRAP-positive area nor TGF-β positive area had a significant difference among the three groups (**Figure 6F-G**). Collectively, these findings suggested that targeted silencing lnc-PMIF in OPCs around bone formation surface could promote bone formation in mice with aging-related osteoporosis.

## Discussion

In the present study, we have uncovered a novel lncRNA-mediated regulatory mechanism that could contribute to the aging-related bone formation reduction. We demonstrated that the aberrantly high expression of lnc-PMIF was responsible for the decreased migration capacity of aged OPCs to bone formation surface during aging. Silencing lnc-PMIF in aged OPCs could facilitate them migrating to bone formation surface for promoting bone formation in aged mice.

During the physiological process of osteoblastogenesis, the OPCs are recruited to the bone remodeling site, where they proliferate, migrate to bone formation surface and undergo osteogenic differentiation toward the functional mature osteoblasts 8, 9. Several previous studies have documented the age-related changes of murine BMSCs *in vitro*, including the increased tendency of adipogenesis^5^, decreased cell proliferative capacity 26 and decreased osteogenic differentiation potential 27, which were considered to account for the decreased bone formation during aging. Interestingly, we did not observe any significant difference in the osteogenic potential of BMSCs *in vitro* between young and aged mice, which is consistent with two previous studies on human mesenchymal OPCs 6, 28. More importantly, we found that both of the* in vitro* and *in vivo* migration capacities of BMSCs from aged mice were remarkably decreased when compared to the BMSCs from young mice. In addition, we also identified a novel lncRNA (*i.e.*, lnc-PMIF) that was consistently upregulated in the aged BMSCs. Surprisingly, the gain-/loss-of-function studies demonstrated that the lnc-PMIF could not only inhibit the migration capacity of OPCs *in vitro* but also suppress their migration to bone formation surface* in vivo*. However, neither overexpression nor silencing of lnc-PMIF could influence the proliferation and osteogenic differentiation of OPCs. All these results demonstrate that the aberrantly high expression of lnc-PMIF in aged OPCs could be responsible for the compromised migration capacity of aged OPCs to bone formation surface, thus contributing to the bone formation reduction during aging **(Figure S11A)**.

Although it was controversial whether BMSCs undergo a decrease of proliferation and differentiation potential *in vivo*, and there were very variable results obtained in different laboratories 29, it has been recently found that BMSCs changed accordingly with the age of an individual 30, 31. Age-dependent loss of cell is not only affected by the external factors and microenvironment, but also associated with the ageing and senescence of progenitor cells themselves 32, 33. The proliferation and differentiation capacity of BMSCs were significantly changed under stress or during aging 34-36. Our current data from high-throughput RNA-Seq, in which a series of migration-related gene downregulated, might support the notion that the cell migration capability also decreased during aging.

The emerging roles of lncRNA in regulating the proliferation, differentiation and senescence of OPCs have been documented in previous researches 32, 37. Interestingly, a recent study has identified the lncRNA *Bmncr* as a key regulator of BMSC fate during skeletal aging by serving as a scaffold to facilitate the interaction of TAZ and ABL, and thus facilitate the assembly of the TAZ and RUNX2/PPARG transcriptional complex for promoting osteogenesis and inhibiting adipogenesis of BMSCs 38. In this study, we found that the lnc-PMIF could interact with the HuR, an RNA-binding protein previously reported to promote cell migration by stabilizing the β-actin mRNA 18, 19, in OPCs. Our in-depth mechanism study demonstrated that lnc-PMIF interacted with HuR through the binding of its center stem-loop structure to the RRM3 domain of HuR, by which it interrupted the interaction of HuR with β-actin mRNA, and therefore leading to the downregulation of β-actin in OPCs **(Figure S11B)**. It is widely recognized that MACF1, crosslinked by F-actin and microtube, was a positive regulator in cell migration 10-13. In this study, we identified lnc-PMIF as a sense-overlapping transcript from Macf1 gene locus and found that lnc-PMIF overexpression had no significant effect on Macf1 while lnc-PMIF silencing could upregulate it, indicating that lnc-PMIF might not directly regulate the expression of Macf1. Consistently, the RNA-pulldown and RIP results showed that Macf1 does not directly interact with neither lnc-PMIF nor HuR. Therefore, there might exist another mechanism of lnc-PMIF in regulating Macf1, which is independent of lnc-PMIF - HuR - β-actin axis. Our study has established a novel role of lncRNA as a key regulator on OPC migration during skeletal aging. In addition, we also identified a functionally conserved human lncRNA ortholog of the murine lnc-PMIF that could inhibit human OPC migration *in vitro*, suggesting that this lncRNA-mediated regulatory mechanism is evolutionarily conserved.

Given the important role of BMSCs as the unique source of OPCs for osteoblastogenesis 39, autologous BMSC transplantation could be a promising anabolic strategy for elder patients with osteoporosis and impaired fracture healing 40. However, the therapeutic effect would be compromised due to the age-related change of BMSCs. In this study, we have shown that silencing lnc-PMIF could facilitate the aged BMSCs migrating to bone formation surface. Importantly, the proliferative capacity and osteogenic differentiation potential of the aged BMSCs were maintained during aging and was not disturbed after silencing lnc-PMIF. More impressively, replenishing these aged BMSCs with lnc-PMIF knockdown could markedly promote bone formation in aged mice. These findings indicate a novel cell-based anabolic therapy for aging-related osteoporosis. Meanwhile, we also demonstrated that targeted delivery of lnc-PMIF siRNA approaching the OPCs around bone formation surface could promote bone formation in aged mice. This finding, on the other hand, hints another novel anabolic strategy for aging-related osteoporosis.

## Materials and Methods

### Animals

The C57BL/6 mice were obtained from the Chinese University of Hong Kong and the Laboratory Animal Center of Air Force Medical University of China and maintained under standard animal housing conditions (12 h light, 12 h dark cycles and free access to food and water) in Hong Kong Baptist University and in Northwestern Polytechnical University. All the experimental procedures were approved by the Committees of Animal Ethics and Experimental Safety of Hong Kong Baptist University (Ref No. (19-120) in DH│HT&A│8│2│6 Pt.1) and by the Institutional Experimental Animal Committee of Northwestern Polytechnical University (No.2017015 & No.2019030).

### DiI labelling and cell intratibial injection

6-month-old C57BL/6J mice male mice will be used as recipients. The mice were anesthetized by injection of pentobarbital sodium, and the left distal femur were gently drilled with a 26 G needle through the patellar tendon. Donor cells were fluorescence labelled by Dil (Beyotime, Jiangsu, China). The Dil-labeled cells (1×10^6^) in 10 μL of α-MEM were injected into the bone cavity of recipient mice through the hole in the femur using a micro-syringe. The recipient mice were sacrificed on 3 days after the intratibial injection. The protocol was modified according to the published reference 41.

### Cell culture

The MC3T3-E1 clone 14 cell line was maintained in Alpha Modified Eagle's Medium (α-MEM, Gibco) containing 10% FBS (Gibco) and 1% penicillin and streptomycin (Gibco). The hFOB1.19 cell line was maintained in DMEM (Gibco) with 10% FBS and 1% penicillin and streptomycin. The primary BMSCs were prepared according to the previously published protocol 42 and maintained in α-MEM (Gibco) with 10% FBS (Corning) and 1% penicillin and streptomycin. The cells were maintained under standard cell culture conditions of 5% CO_2_ and 95% humidity and were not used beyond passage 25. For the experiments, confluent cells were removed using 0.25% trypsin containing 10 mM EDTA, resuspended in antibiotic-free growth medium and plated onto six-well plates at a density of 200,000 cells per well.

### DiI labelling and cell intratibial injection

6-month-old C57BL/6J mice male mice will be used as recipients. The mice were anesthetized by injection of pentobarbital sodium, and the left distal femur were gently drilled with a 26 G needle through the patellar tendon. Donor cells were fluorescence labelled by Dil (Beyotime, Jiangsu, China). The Dil-labeled cells (1×10^6^) in 10 μL of α-MEM were injected into the bone cavity of recipient mice through the hole in the femur using a micro-syringe. The recipient mice were sacrificed on 3 days after the intratibial injection. The protocol was modified according to the published reference 41.

### Cell proliferation assay

Cells were seed at a concentration of 1X10^4^ cell/cm^2^ in 24-well plates and maintained under standard cell culture conditions of 5% CO_2_ and 95% humidity. At 1, 2, 3 day, the cells were completely digested by using 0.25% trypsin containing 10 mM EDTA and transferred into Vi-Cell respectively. Cell viability and cell count were detected by Vi-CELL™ Cell Counter (Beckman Coulter).

### Cell transfection

Transient transfection of cells with siRNA or DNA plasmids was performed in 24-well plates using Lipofectamine 3000 reagent (Invitrogen). HuR expression plasmid (500 ng per well), empty plasmid (500 ng per well), siRNA control (30 nM) and siRNA (si-HuR/si-PMIF) (30 nM) were transfected into cells in culture medium and then harvested for further detection.

### Stable Lnc-PMIF knockdown and overexpression

(1) As for lnc-PMIF knockdown, BLOCK-iT Lentiviral RNAi Expression System (Invitrogen) was used to generate the lentiviral lnc-PMIF shRNA. Briefly, the shRNA sequences were cloned to the pENTR^TM^/U6 Entry vector. Then the pLenti6/ BLOCK-iT^TM^ expression construct was then generated by recombination with pLenti puro/H1TO/BLOCK-iT/DEST vector. The sequences for shRNA against lnc-PMIF were shown as follows: shRNA targeted to 718-736 of the lnc-PMIF (CCTTAGGTGCCTTTAGAAA). (2) As for lnc-PMIF overexpression, the lnc-PMIF sequences were cloned into pENTR/D-TOPO MCS vector. Then the engineered plasmid containing lnc-PMIF sequence was used for recombined with pLenti puro/TO/V5/DEST vector and packaged by ViraPower^TM^ Packaging Mix, for lentivirus-mediated overexpression of interest genes. The resulting plasmid was used for lnc-PMIF overexpression in cells.

### Migration assays

(1) Wound healing assay: Cell monolayers were wounded using a sterile 10 μL pipette tip. Phase contrast images were taken immediately after wounding and at 18 h post-stimulation using a microscope (Leica, DMI3000B, German) connected to a video camera (Sony Corporation, Tokyo, Japan). To measure migration, wound area was quantified using image analysis software (Image J, NIH, USA) and expressed as normalized width of wound closure. (2) Transwell assay: Cell migration was determined using a 6.5 mm transwell chamber with an 8 μm pore size (Corning Costar Inc., Corning, NY, USA). Cells were seeded at a density of 3×10^4^ in the upper compartment of each chamber and after 16 h of serum deprivation, the serum was added in the bottom chamber. After 6 h, cells were removed from the upper membrane surface by a cotton swab and washed with PBS. Migration values were determined by counting three fields per chamber after fixing the membrane in 4% paraformaldehyde and staining with Hematoxylin Staining Solution (Beyotime, Jiangsu, China).

### MTT - cell proliferation assay

The Vybrant® MTT Cell Proliferation Assay Kit was used. Firstly, a 12 mM MTT stock solution was prepared by adding 1 mL of sterile PBS to one 5 mg vial of MTT (Component A). Then, 10 mL of 0.01 M HCl was added to one tube containing 1 gm of SDS (Component B). The isolated osteogenic cells were washed with 100 µL of fresh medium, added with 10 µL of the 12 mM MTT stock solution and incubated at 37 °C for 4 h. Then, 100 µL of the SDS-HCl solution was added to each well, mixed thoroughly using the pipette and incubated at 37 °C for 4 h in a humidified chamber. Then, each sample was mixed again using a pipette and the absorbance was read at 570 nm.

### Northern blot

Total RNA extracted from mouse and human cells were separated by PAGE (7M urea) on 6% polyacrylamide gels and transferred to a plus positively charged nylon membrane (Invitrogen). Labelled Lnc-PMIF probes were synthesized in RiboBio Company (Guangzhou, China). RNA blots were hybridized in UV Hybridization Incubator (UVP HB1000, USA) at 68 °C overnight, washed twice (5 min) with 2X saline sodium citrate (SSC) / 0.1% SDS wash buffer at 68 °C, followed by stringent washes (2X30 min) with 0.1×SSC/0.1% SDS wash buffer at 68 °C. Blots were visualized using Bio-Rad ChemiDoc XRS+ (Bio-Rad).

### RNA isolation and real-time PCR analysis

Total RNA was extracted from frozen tissue with a polytron homogenizer (Prima PB100, Shanghai, China) or directly from cells by using TRIzol reagent (Invitrogen, Rockville, MD). Total RNA was used as a template for double-stranded cDNA synthesis (PrimeScript™ RT reagent Kit, Takara, Dalian, China). The SYBR® Premix Ex Taq™ II (Takara, Dalian, China) was applied for the quantitative RT-PCR. GAPDH were used as endogenous controls for normalization. All the primer sequences were listed in Supplemental Table S1. The relative fold changes of candidate genes were analyzed by using 2^-ΔΔCT^ method 43.

### Rapid Amplification of cDNA Ends (RACE)

The transcription start/end site of lnc-PMIF were determined by using 3' and 5'RACE kits based on the manufacturer's instructions (TaKaRa). All RACE primers are listed in Table S2.

### RNA Fluorescence *in situ* Hybridization (RNA-FISH)

Fluorescence-conjugated lnc-PMIF probes (5′-ACTCAGCACAGCAACACTAGCT-3′) were synthesized in RiboBio Company (Guangzhou, China) and the lnc-PMIF was detected by FISH Kit (RN: R11060.4, RiboBio, Guangzhou, China) according to the manufacturer's instructions. Images were obtained with a Nikon 80i Fluorescence Microscope.

### RNA pulldown + MS/ WB

The RNA pull-down assay was conducted as described 44. Sense and antisense strands of PMIF cDNA were synthesized by GenePharma (Shanghai, China), annealed and cloned into pGEM-T in front of a cloning site into which was inserted sequences encoding wild-type or truncated versions of PMIF; truncations were made at roughly 250-590 bp intervals from either the 5′ or 3′ end. Biotin-labeled PMIF and truncated RNAs were obtained by transcription *in vitro* (T7 RNA Polymerase, 10× Transcription buffer, Roche), labeled with biotin RNA labeling mix (Roche), and then incubated with nuclear extracts separated from MC3T3-E1 cells. Pulldown components were separated by SDS-PAGE and silver staining. Finally, differential bands were analyzed by western blotting or mass spectrometry (MS) as previous describe 45. Western blotting in the RNA pull-down assay was performed with rabbit anti-HuR antibody (1:1000; ab200342, Abcam, USA) and anti-LMNA (Lamin A/C) antibody (1:1,000; PB0307, Boster, China).

### RNA-binding-protein immunoprecipitation assay (RIP)

Isolation of MC3T3-E1 cell was lysed in polysome lysis buffer (100 mM of KCl, 5 mM of MgCl_2_, 10 mM of HEPES [pH 7.0], 0.5% NP-40, and 1 mM of dithiothreitol), supplemented with complete protease inhibitors (Beyotime, Haimen, China) and Recombinant RNase Inhibitor (TaKaRa, Dalian, China) for a 30 min ice bath and centrifugalization (12,000g for 15 min). Supernatants were precleared with Protein A/G beads and incubated with rabbit anti-HuR antibody (Abcam, ab200342, 1:1,000) or rabbit IgG (Beyotime, Haimen, China) at 4 °C overnight. Protein A/G beads were then added and incubated at 4 °C for 4 h. Finally, binding RNA was isolated by phenol-chloroform-isoamyl alcohol for RT-qPCR.

### RNA electrophoretic mobility shift assay (EMSA)

RNA probes were synthesized from linearized pcDNA3.1-PMIF and pcDNA3.1-ACTB using MAXIscript™ T7 Transcription Kit (Invitrogen), and labeled with biotin RNA labeling mix (Roche) following the manufacturer's instructions. The plasmid of wildtype HuR and its fragment deletion mutations were synthesized from pcDNA3.1-HuR plasmid (GenePharma, Shanghai, China). RNA EMSA experiments were conducted according to the manufacturer's protocol with LightShift™ Chemiluminescent RNA EMSA Kit (Thermo Fisher Scientific). HuR (WT/mutant) was incubated with or without unlabeled probe for competitive reaction and anti-Flag for supershift at 25 °C for 20 min in a reaction buffer. Then biotin-labeled probe was added into the reaction system and incubated for 20 min at 25 °C. Unlabeled probes at indicated concentrations were used for competition experiments. The reactions were then loaded onto a 1% 0.5× TBE-agarose gel and transferred to a positively charged nylon membrane (Invitrogen). The membrane was then cross-linked by exposure to UV (UVP HB1000, USA), incubated with HRP-conjugated streptavidin and visualized with ECL reagents. The dissociation constant (Kd) was calculated as the concentration of unlabeled probe when half of the labeled probe was dissociated from the complex with HuR.

### RNA Sequencing (RNA-Seq)

The high-throughput RNA-Seq experiments were performed by Annoroad Gene Technology (Beijing, China). Briefly, total RNAs was extracted from cells by TRIzol Reagent (Life Technologies). Then the RNA samples were evaluated for degradation and impurities by 1% agarose electrophoresis. The RNA purity was determined using a NanoPhotometer (IMPLEN, CA, USA). An Agilent 2100 Bioanalyzer and Agilent RNA 6000 Nano Kit were used to assess RNA concentration and integrity (Agilent Technologies, CA, United States). The samples were sequenced and samples with RNA Integrity Number (RIN) scores > 9.5 were used in subsequent analyses. The mouse genome was selected as the reference genome in this study. High -throughput sequencing was performed by Annoroad technologies (Beijing, China). The lncRNA library was constructed from total RNA (2 μg) using different index tags with the next Ultra directional RNA library prep Kit for Illumina (NEB, Ipswich, USA) according to the manufacturer's instructions. Data quality control involved removal of contaminated reads, low quality reads, reads containing N ratios >5%, and reads that matched the ribosomal RNA. The RNA-seq data filtered for each sample was compared with the genome by HiSAT2. FPKM was used to estimate gene expression levels quantitatively.

### Western blot

Tissues or cells were lysed in in a buffer containing 50 mM Tris (pH 7.5), 150 mM NaCl, 0.5% NP40 (Beyotime, China), and protease inhibitors (TaKaRa, China). Proteins in lysates were resolved by SDS-PAGE and then transferred to polyvinylidene difluoride membranes. The membranes were blocked with 5% BSA for 1 h at room temperature and incubated with primary antibody recognizing MACF1 (Abcam, ab117418, 1:1000), Runx2 (Abcam, ab23981, 1:500), Osteocalcin (Abcam, ab93876, 1:500), CollagenⅠ (Abcam, ab34710, 1:1,000) , HuR (Abcam, ab200342, 1:1,000), β-Actin (Boster, BM3873, 1:1,000), CBF-A (GeneTex, GTX121922, 1:1,000) , Lamin A/C (Boster, PB0307, 1:1,000) or Gapdh (Boster, BM1623, 1:1,000) at 4 °C. Incubation with secondary horseradish peroxidase-labelled antibody was carried out for 1 h at room temperature.

### Alizarin red staining

Cells were fixed in 70% ice-cold ethanol for 1 h and rinsed with double-distilled H_2_O. Cells were stained with 40 mM Alizarin Red S (Sigma), pH 4.0, for 15 min with gentle agitation. Cells were rinsed five times with double-distilled H_2_O and then rinsed for 15 min with 1× PBS while gently agitating.

### ALP staining

Alkaline phosphatase staining was monitored using an Alkaline Phosphatase Assay Kit (C3206, Beyotime). Cells were fixed by immersion 4% paraformaldehyde (PFA) solution for 10 min and rinsed in PBS for 5 min 3 times. The samples were then placed in an alkaline phosphatase staining solution (BCIP/NBT solution) for 30 min. The whole procedure was protected from light. After discarding the solution, cells were rinsed in deionized water 2 min 2 times.

### Flow cytometry

Flow cytometry was performed with a Becton Dickinson flow cytometer to quantify the cell cycle. In brief, cells were washed twice with phosphate-buffered saline (PBS), trypsinated, and resuspended in PBS-2% FCS-20 mM EDTA. Analysis was performed in a Becton Dickinson flow cytometer with appropriate filter settings. The data were quantified by using the CellQuest software package.

### Fluorescent activated cell sorting (FACS)

The FACS samples were prepared in a RNase-free environment. Cells were harvested from the cancellous bone region of long bones as follows. The epiphysis was removed to dissect the metaphyseal bone region extending about 5 mm from the growth plate, which was crushed with mortar and pestle in FACS Buffer, followed by digestion with 0.25% collagenase type I (Thermo Fisher, 17100017) in PBS for 30 min at 37 °C with gentle shaking. The cells were then centrifuged and re-suspended with FACS Buffer and filtered with a 70-μm cell strainer. The cells were fixed in 0.01% formaldehyde for 10 min, washed with PBS, and incubated with Alexa Fluor 594-conjugated Mouse monoclonal anti-Gli1 antibody (1:10 dilution; Santa Cruz sc-515751 AF594) diluted in cell permeabilization medium (Abcam, ab185917) for 1 h at room temperature. Thereafter, the cells were washed in PBS, re-suspended in FACS buffer and subjected for cell sorting by the BD FACSAria™ III cell sorter. The Gli1+ cells were collected for RNA isolation and the subsequent reverse transcription PCR and QPCR analysis.

### Fluorescent immunohistochemistry

The proximal tibia from the mice were fixed with 4% PFA before embedding in optimum cutting temperature (OCT, Tissue-Tek, USA). Then, the series-frozen sections (5 μm) were prepared in a cryostat (Thermo Scientific™ CryoStar™ NX70) at -24 ºC. Undecalcified sections were generated using the Kawamoto's film method.

For immunostaining, sections were incubated with blocking buffer containing 10% donkey serum and 0.15% Triton X-100 (Abcam # ab7475) for 1 h followed by incubating with primary antibodies Rabbit monoclonal anti-Runx2 antibody (1:1000, abcam, ab23981) at 4 °C overnight. Thereafter, the sections were washed and incubated with Alexa Fluor 647-conjugated fluorescent secondary antibodies (1:400, Thermo Fisher, A-31573) for 1 h. After three wash in PBS, the sections were mounted by cover slides with Fluoroshield Mounting Medium with DAPI (Abcam, ab104139). Dil^+^ cell number per bone perimeter (Dil^+^.N/B.Pm) The immunostained section were made using professional image analysis software (Image J, NIH) under fluorescence microscope (Leica image analysis system, Q500MC).

For immunohistochemical (IHC) staining, a Pierce™ Peroxidase IHC Detection Kit (Thermo Fisher, 36000) was used. Sections were incubated with blocking buffer containing 10% donkey serum and 0.3% Triton X-100 for 1 h followed by incubating with primary antibodies Rabbit monoclonal antibody (Rb mAb) of MACF1 (1:250, abcam, ab117418), Rb mAb of Dynein (1:250, GeneTex, GTX120114) and Rb mAb of TGF-beta3 (1:400, abcam, ab15537) at 4 °C overnight. Thereafter, the sections were washed and incubated with HRP-conjugated secondary antibodies (1:1000, Thermo Fisher, 31466) for 1 h. Following three washes in PBS, the sections were developed for color reaction using DAB/Metal solution and hematoxylin counterstain. The immunostained section were made using professional image analysis software (Image J, NIH) under microscope (Leica, DMI3000B, German).

### Fluorescent immunocytochemistry

For immunofluorescent staining, cells were fixed with either 4% PFA for 20 min at room temperature followed by permeabilization with 0.2% Triton X-100 for 2 min. Next, samples were blocked with 1% BSA diluted in 1× PBS supplemented with 0.05% Tween-20 for 45 min and sequentially incubated with primary anti-β-Actin antibody (1:1,000, Abcam, ab9574) at 4 ºC overnight and fluorescently labeled secondary antibodies for 1 h. Finally, samples were washed, dried and mounted in DAPI solution (Beyotime).

### Laser Captured Micro-dissection (LCM)

The LCM samples were prepared in a RNase-free environment. Briefly, the tibias fixed by 4% PFA before embedding in OCT. Then, the series frozen sections (5 µm) were prepared using the Kawamoto's film method in a cryostat (Thermo Scientific™ CryoStar™ NX70) at -24 ºC. The sections were mounted on LMD films (SECTION-LAB Co. Ltd.). The cryosections were then incubated with Alexa Fluor 594-conjugated Mouse monoclonal anti-Gli1 antibody (1:50 dilution; Santa Cruz sc-515751 AF594) for 1h at room temperature. After brief rinsing in RNase-free water, the sections were air-dried. Gil1-positive cells around the calcein labeled bone surface were isolated by microdissection with an upgraded laser pressure catapulting microdissection system (P.A.L.M.) using a pulsed 355 nm diode laser in the Leica LMD 7000 Laser Microdissection System. About 150~200 identified cells were collected in reaction tube containing 5 µL lysis buffer for total RNA extraction and the subsequent reverse transcription PCR and QPCR analysis.

### Bone histomorphometry

The distal femur was dehydrated in graded concentrations of ethanol and embedded without decalcification in the modified methyl methacrylate using our previously established protocol 46. After dehydration, frontal sections for trabecular bone were obtained from the distal femur at a thickness of 5 μm with Leica SM2500E microtome (Leica Microsystems). Fluorescence micrographs for the calein in the bone sections was captured by a fluorescence microscope (Leica image analysis system). Bone dynamic histomorphometric analyses for MAR (mineralizing apposition rate) and BFR/BS (bone formation rate/bone surface) were made using professional image analysis software (Image J, NIH) under fluorescence microscope (Leica image analysis system, Q500MC). The bone histomorphometric parameters were calculated and expressed according to the standardized nomenclature for bone histomorphometry 46.

### MicroCT analysis

The femur was scanned by the microCT system (version 6.5, viva CT40, SCANCO Medical, Switzerland) and the left distal femoral metaphysis was analyzed according to the previously published protocols 24, 47, 48. Briefly, the femur was dissected from the mice free of soft tissue, fixed overnight in 70% ethanol and analyzed by microCT. Images of femurs were reconstructed and calibrated at the isotropic voxel size of 10.5 μm, respectively (70 kVp, 114 μA, 200 ms integration time, 260 thresholds, 1200 mg HA/cm^3^). Using the Scanco evaluation software, regions of interest (ROIs) were defined for both cortical and trabecular parameters. For the left distal femoral metaphysis, the entire femora were reoriented with the mid-diaphysis parallel to the z-axis, and bone length were measured as the distance between the most proximal and distal transverse plans containing the femur. Beginning from the most proximal aspect of the growth plate, the trabeculae region on 100 consecutive slices were selected. The trabeculae were analyzed by manually contouring excluding the cortical bone for three-dimensional reconstruction (sigma = 1.2, supports = 2 and threshold = 200) to calculate the following trabeculae parameters: Tb.BV/TV (trabecular bone volume per total volume), Tb.vBMD (trabecular volumetric bone mineral density), Tb.Th (trabecular thickness) and Tb.Sp (trabecular separation).

## Supplementary Material

Supplementary figures and tables.

## Abbreviations

AdMSCs: adipose-derived mesenchymal stem cells
ALP: Alkaline phosphatase
BFR: bone formation rate
BMD: bone mineral density
BMSCs: bone marrow mesenchymal stem cells
BV/TV: bone volume per total volume
EMSA: electrophoretic mobility shift assay
FISH: fluorescence *in situ* hybridization
HSPCs: hemopoietic stem/progenitor cells
HuR: human antigen R
KD: knockdown
kDa: kilo Dalton
MACF1: Microtubule actin crosslinking factor 1
MAR: mineralizing apposition rate
MF: microfilaments
MS: Mass Spectrometry
MSPCs: mesenchymal stem/progenitor cells
OE: overexpression
OPCs: osteoprogenitor cells
PMIF: postulated migration inhibitory factor
QPCR: quantitative RT-PCR
RACE: Rapid Amplification of cDNA Ends
RIP: RNA immunoprecipitation
RRM: RNA recognition motif
siRNA: small interfering RNA
WB: western blot
WT: wildtype
