# Supplementary material for: Targeting long noncoding RNA PMIF facilitates osteoprogenitor cells migrating to bone formation surface to promote bone formation during aging
Source: Theranostics. 2021 Mar 20;11(11):5585–604. doi: 10.7150/thno.54477 (PMC8039942; doi:10.7150/thno.54477)
Supplement: Supplementary file 1 — Supplementary figures and tables. [file thnov11p5585s1.pdf]

Supplementary material

Targeting long noncoding RNA PMIF facilitates osteoprogenitor cells migrating to bone formation surface to promote bone formation during aging

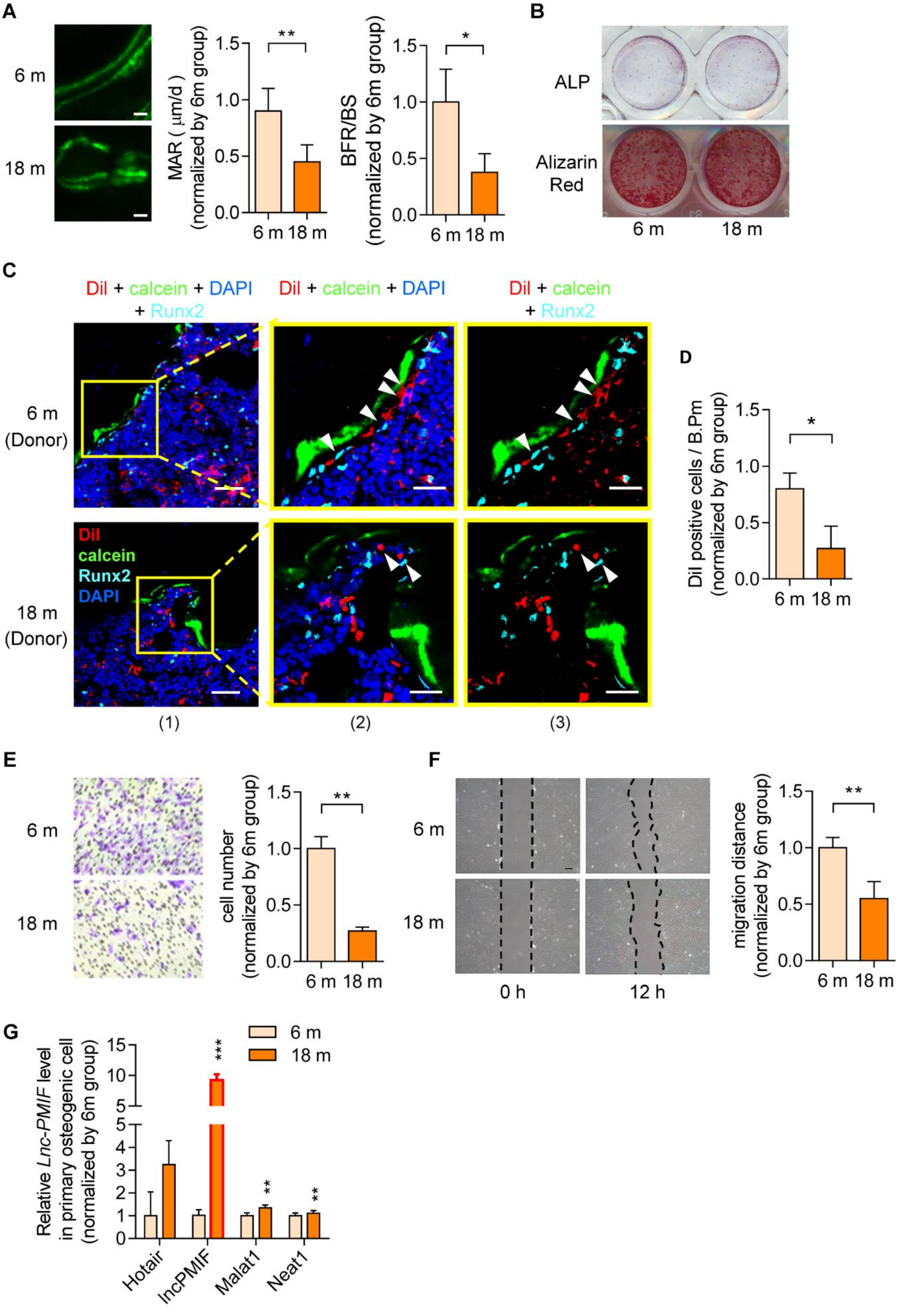
A
6 m
18 m
MAR (μm/d)
(normalized by 6m group)
**
6 m 18 m
BFR/BS
(normalized by 6m group)
*
6 m 18 m
B
ALP
Alizarin Red
6 m
18 m
C
Dil + calcein + DAPI + Runx2
Dil + calcein + DAPI
Dil + calcein + Runx2
6 m (Donor)
18 m (Donor)
Dil
calcein
Runx2
DAPI
(1)
(2)
(3)
D
Dil positive cells / B.Pm
(normalized by 6m group)
*
6 m 18 m
E
6 m
18 m
cell number
(normalized by 6m group)
**
6 m 18 m
F
6 m
18 m
0 h
12 h
migration distance
(normalized by 6m group)
**
6 m 18 m
G
Relative Lnc-PMIF level
in primary osteogenic cell
(normalized by 6m group)
6 m
18 m
***
**
**
Hotair
lncPMIF
Malat1
Neat1

Figure S1. Decreased bone formation accompanied by reduced migration of OPCs to bone formation surface and elevated lnc-PMIF expression in OPCs in female mice during aging. (A) Dynamic bone histomorphometry of distal femoral metaphysis of young (6-month-old, 6 m) and aged (18-month-old, 18 m) mice. Top panel: the representative fluorescent images of new bone formation revealed by double calcein labeling. Bottom panel: the dynamic bone histomorphometric parameters (MAR and BFR/BS). **(B)** Representative images of ALP staining in BMSCs after 7 days of osteogenic induction (top) and Alizarin Red S staining in BMSCs after 14 days of osteogenic induction (bottom). Scale bar: 100 μm. **(C)** Representative confocal images of tibia metaphysis showing the Dil-labeled cells (red) and Runx2-expressing cells (light blue) on and around the calcein-labeled bone formation surface (green) at 3 days after injection. Cell nucleus were stained by DAPI (dark blue). Scale bar: 100 μm (left panel) and 25 μm (middle and right panels). Arrow heads indicate the Dil-positive cells at the bone formation surface. **(D)** The average number of Dil-labeled cell approaching bone formation surface. n=3~4 mice per group. **(E)** Transwell migration assay of BMSCs *in vitro*. Left: Representative images of the migrated cells. Right: the number of migrated cells. **(F)** Wound healing assay of BMSCs *in vitro*. Left: Representative images of the miagrated cells. Right: the migration distance**. (G)** QPCR analysis of the expression of Hotair, lncPMIF, Malat1 and Neat1 in BMSCs***. Note:*** BMSCs were isolated from the young and aged mice, respectively. For *in vitro* assay the experiments were conducted in triplicates. For *in vivo* assay, n=6 mice per group unless specifically annotated. All data were expressed as mean ± SD. *$P < 0.05$, **$P < 0.01$, ***$P < 0.01$ by Student's *t*-test.

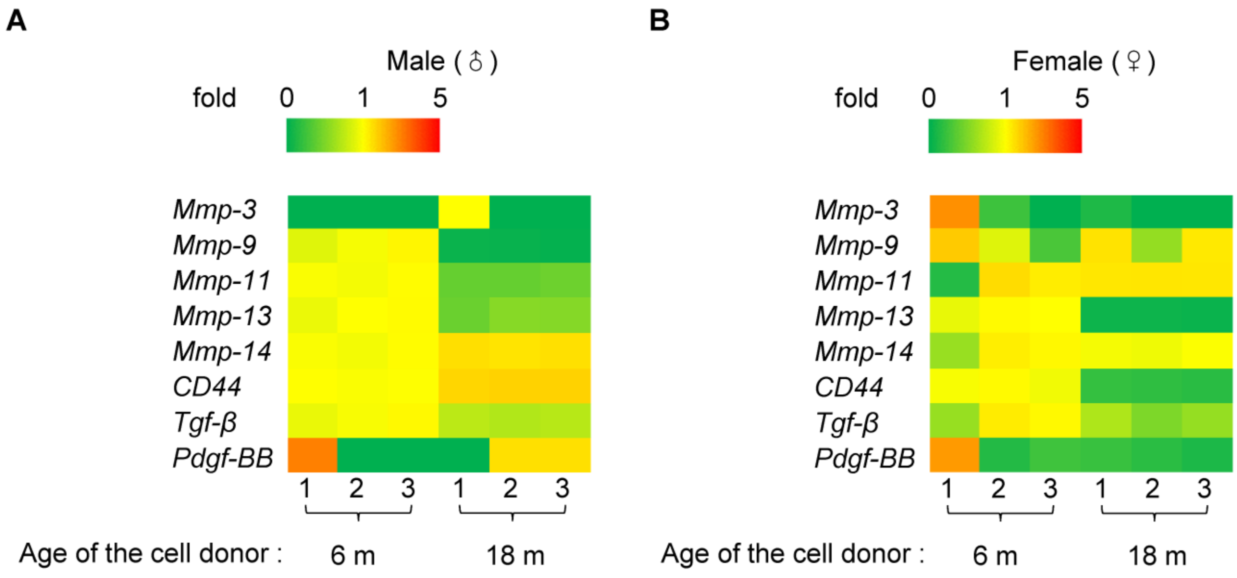

Figure S2. Heat map of the genes encoding migration-related proteins in BMSCs isolated from male and female mice during aging by RNA-Seq. n=3 mice per group

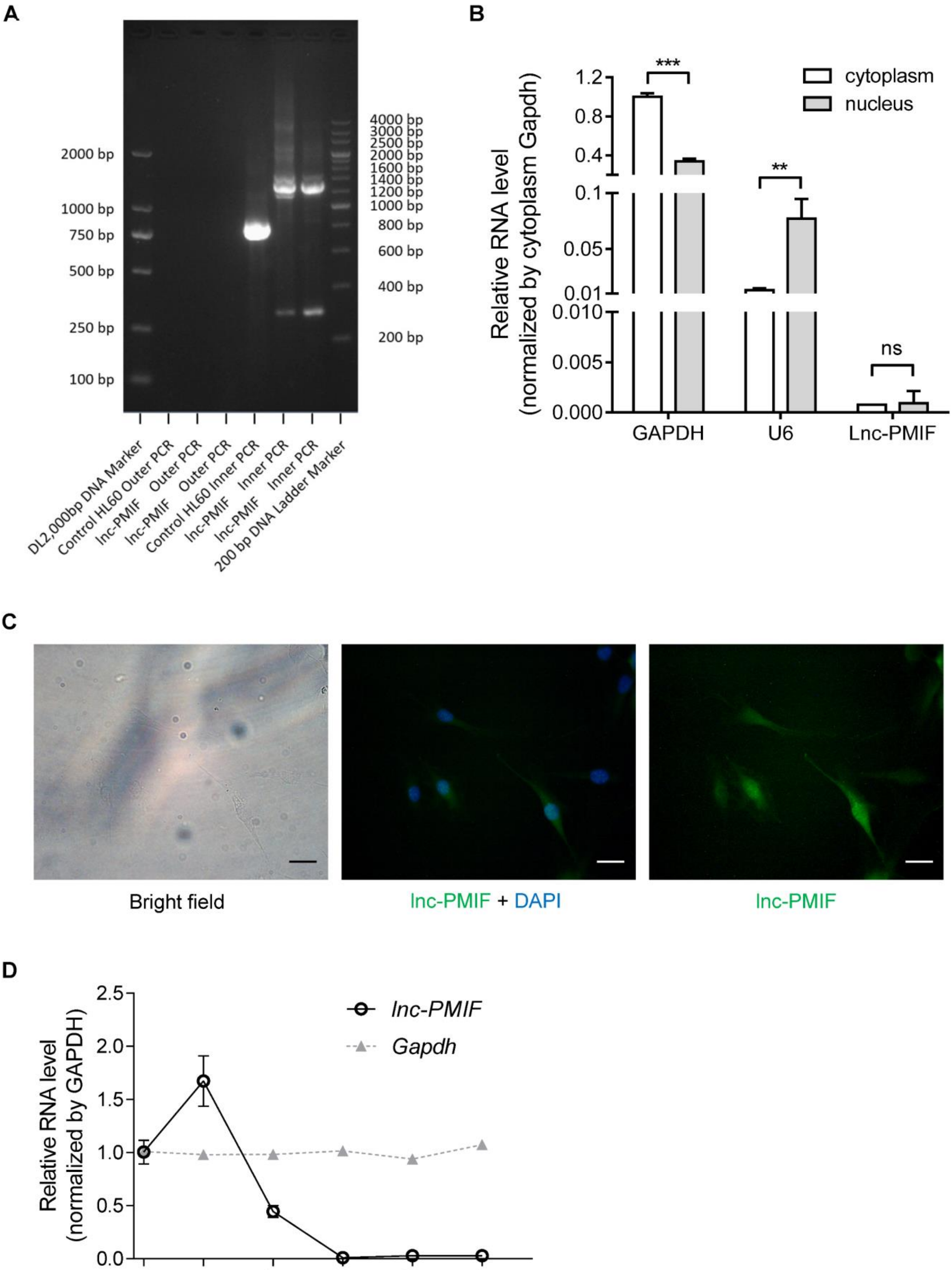
A
2000 bp
1000 bp
750 bp
500 bp
250 bp
100 bp
4000 bp
3000 bp
2500 bp
2000 bp
1600 bp
1400 bp
1200 bp
1000 bp
800 bp
600 bp
400 bp
200 bp
DL2,000bp DNA Marker
Control HL60 Outer PCR
lnc-PMIF Outer PCR
lnc-PMIF Outer PCR
Control HL60 Inner PCR
lnc-PMIF Inner PCR
lnc-PMIF Inner PCR
200 bp DNA Ladder Marker
B
Relative RNA level
(normalized by cytoplasm Gapdh)
1.2
0.8
0.4
0.1
0.05
0.01
0.010
0.005
0.000
***
**
ns
cytoplasm
nucleus
GAPDH
U6
Lnc-PMIF
C
Bright field
lnc-PMIF + DAPI
lnc-PMIF
D
Relative RNA level
(normalized by GAPDH)
2.5
2.0
1.5
1.0
0.5
0.0
lnc-PMIF
Gapdh
1
7
14
21
28
35
Day after mineralization induced

Figure S3 Identification of the full length and cellular distribution of lnc-PMIF. (A) Representative images of agarose gel electrophoresis showing the lnc-PMIF amplificated by 3'RACE PCR. **(B)** QPCR analysis showing the expression of lnc-PMIF, GAPDH and U6 in nuclei and cytoplasm, respectively. **(C)** Representative images of the expression of lnc-PMIF in nuclei and cytoplasm by fluorescence *in situ* hybridization analysis. **(D)** QPCR analysis of lnc-PMIF expression during osteoblast differentiation of MC3T3-E1 cells. ***Note:*** All experiments were conducted in triplicates. All data were expressed as mean ± SD. **$P < 0.01$, ***$P < 0.001$ by Student's *t*-test.

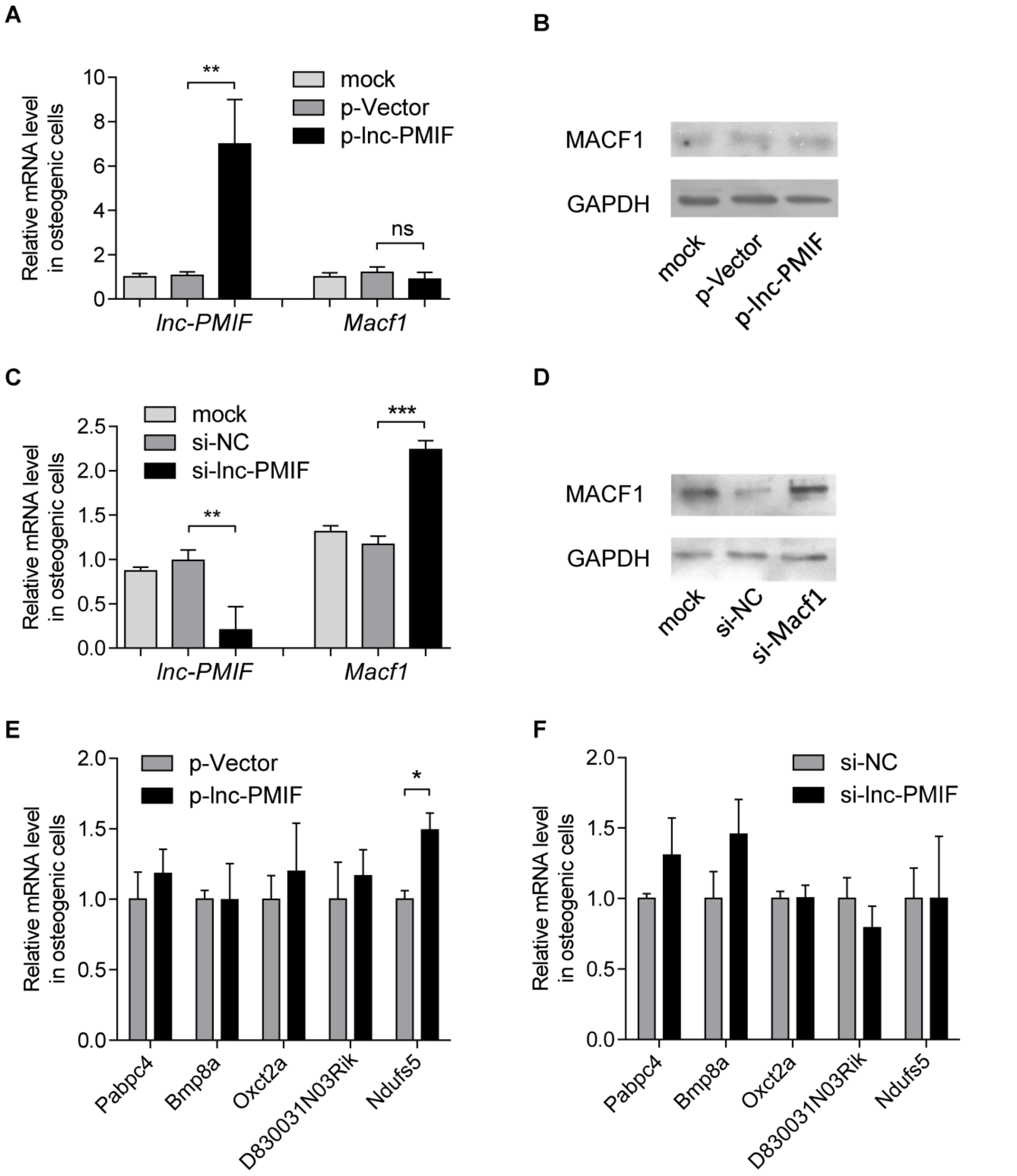

Figure S4 The effect of lnc-PMIF on Macf1 expression in OPCs. (A, **B)** QPCR and Western blot (WB) analysis of the Macf1 expression level after the MC3T3-E1 cells were transfected with plasmid of lnc-PMIF. Mock: transfection reagent only; p-Vector: empty plasmid; p-lnc-PMIF: lnc-PMIF expression plasmid. **(C**, **D)** QPCR and WB analysis of the Macf1 expression level after the MC3T3-E1 cells were transfected with siRNA of lnc-PMIF. si-NC: siRNA of Negative Control (NC); si-lnc-PMIF: siRNA of lnc-PMIF. **(E**, **F)** QPCR analysis of coding and noncoding transcripts related with lnc-PMIF and Macf1 after the MC3T3-E1 cells were transfected with plasmid / siRNA of lnc-PMIF. ***Note:*** All experiments were conducted in triplicates. All data were expressed as mean ± SD. **$P < 0.01$, ***$P < 0.001$ by Student's *t*-test.

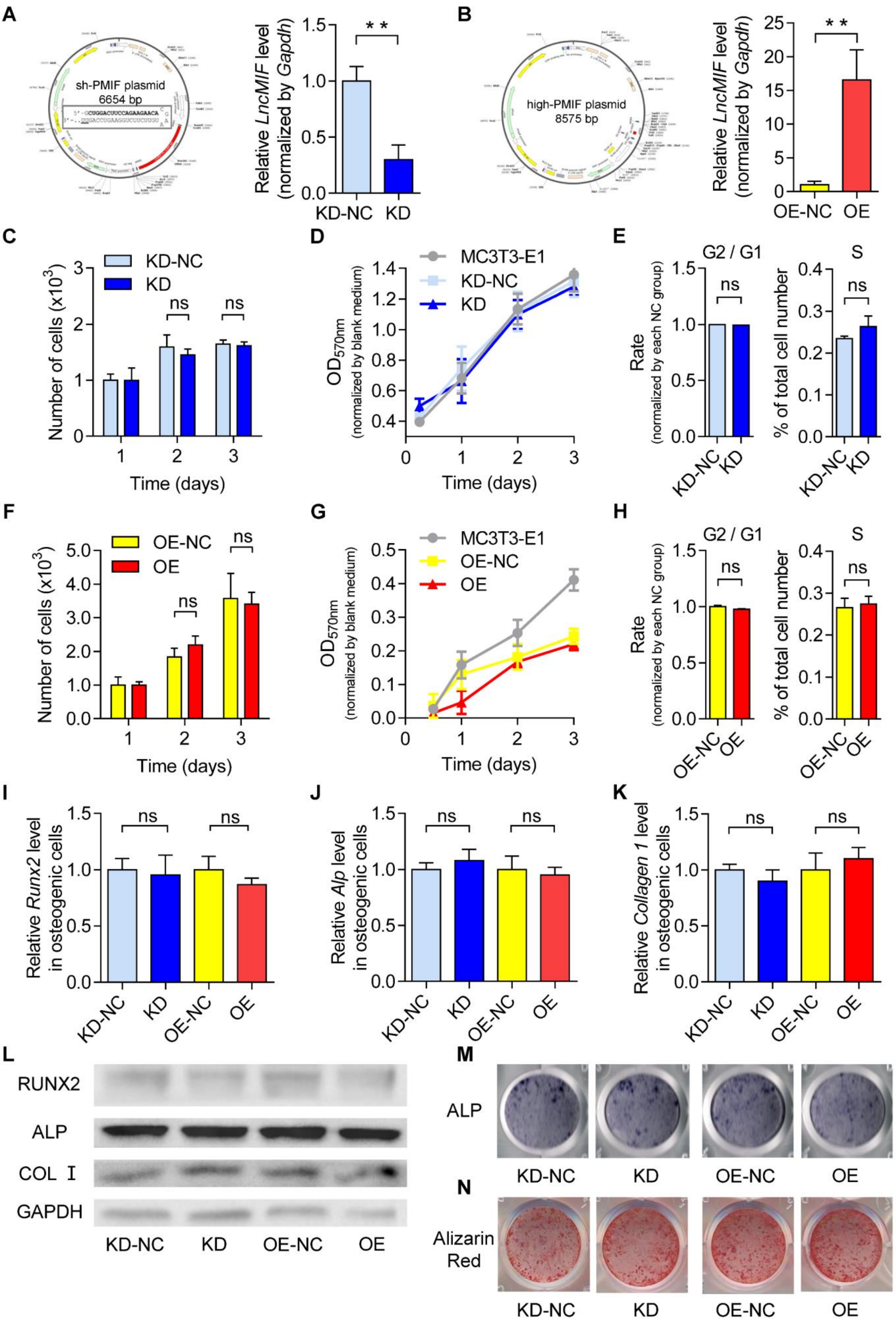

A
sh-PMIF plasmid
6654 bp
Relative LncMIF level (normalized by Gapdh)
KD-NC
KD
B
high-PMIF plasmid
8575 bp
Relative LncMIF level (normalized by Gapdh)
OE-NC
OE
C
Number of cells (x10^3)
Time (days)
D
MC3T3-E1
OD570nm (normalized by blank medium)
E
G2 / G1
S
Rate (normalized by each NC group)
% of total cell number
F
G
H
I
Relative Runx2 level in osteogenic cells
J
Relative Alp level in osteogenic cells
K
Relative Collagen 1 level in osteogenic cells
L
RUNX2
ALP
COL I
GAPDH
M
N
Alizarin Red
ns

Figure S5 The effect of lnc-PMIF gain- and loss-of-function on the proliferation and osteogenic potential of OPCs. (A) The construction of lnc-PMIF knockdown MC3T3-E1 cells. Left: schematic diagram of the lnc-PMIF shRNA-expressing plasmid. Right: QPCR analysis of the lnc-PMIF expression in KD and KD-NC cells. **(B)** The construction of lnc-PMIF overexpressing MC3T3-E1 cells. Left: schematic diagram of the lnc-PMIF-expressing plasmid. Right: QPCR analysis of the lnc-PMIF expression in OE and OE-NC cells. **(C**, **F)** The cell number of MC3T3-E1 cells in different groups during *in vitro* proliferation. **(D**, **G)** The cell viability of MC3T3-E1 cells in different groups during *in vitro* proliferation by MTT assay. **(E**, **H)** The ratio of MC3T3-E1 cells at G2 phase to the cells at G1 phase and the percentage of MC3T3-E1 cells at S phase in different groups during *in vitro* proliferation by Flow cytometry analysis. **(I-K)** The mRNA expression of osteoblast-specific markers Runx2, ALP and Collagen Type I in MC3T3-E1 cells in different groups after osteogenic induction by QPCR analysis. **(L)** The protein expression of osteoblast-specific markers Runx2, ALP and Collagen Type I in MC3T3-E1 cells in different groups after osteogenic induction by western blot. **(M)** Representative images of ALP activity in MC3T3-E1 cells in different groups at 7 days after osteogenic induction by ALP staining. **(N)** Representative images of calcium mineral deposition in MC3T3-E1 cells in different groups at 14 days after osteogenic induction by Alizarin Red S staining. Scale bar: 100 μm. ***Note:*** All experiments were conducted in triplicates. All data were expressed as mean ± SD. ns: not statistically significant, $^{**}P < 0.01$ by Student's *t*-test.

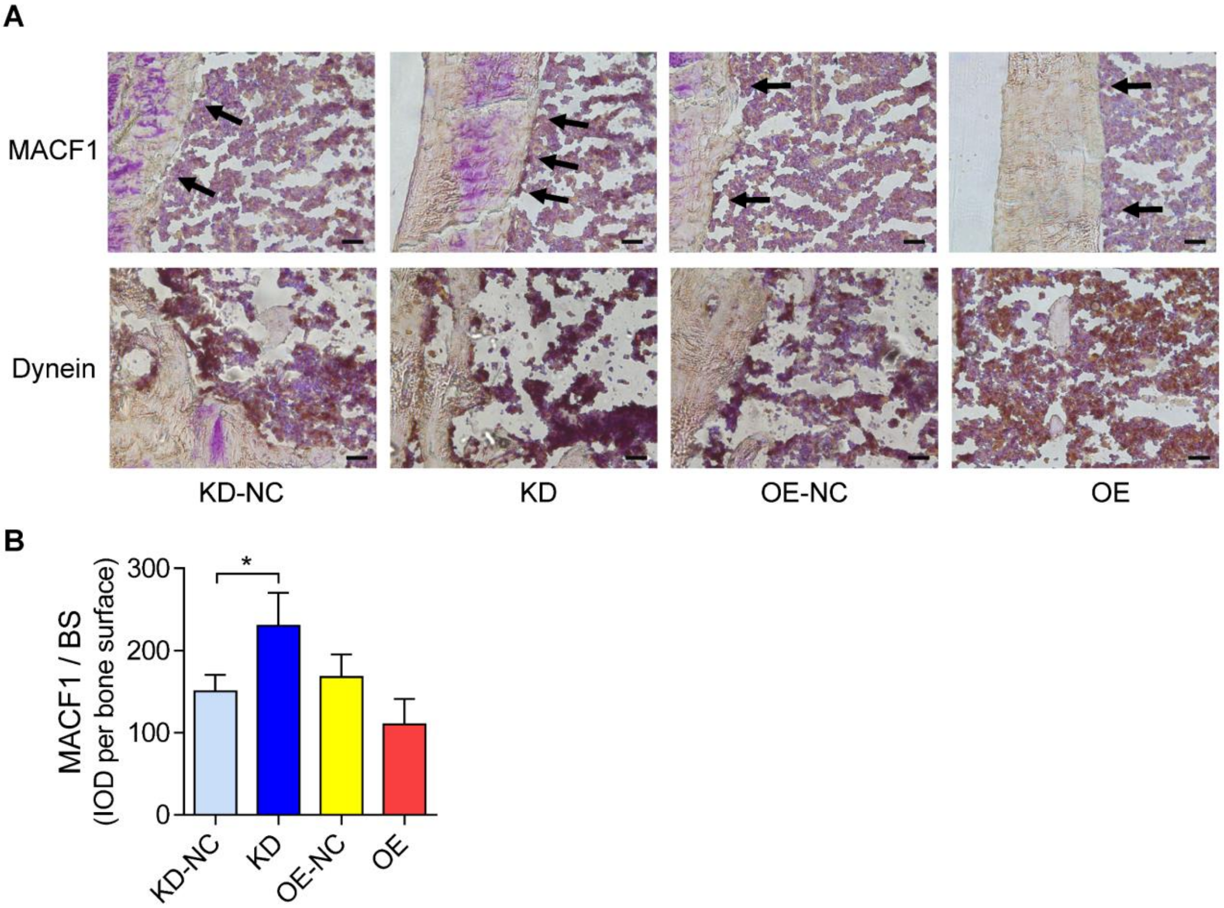

Figure S6. Immunohistochemical (IHC) staining of MACF1 and Dynein in the mice received intra-bone-marrow cell injection (refer to Figure 2)**. (A)** Representative microscopy images of tibia metaphysis showing the IHC staining of MACF1 (top) and Dynein (bottom) on and around the bone surface at 3 days after injection. Cell nucleus were stained by hematoxylin. Scale bar: 50 μm. Arrow heads indicate the MACF1 positive cells at the bone surface (BS). **(B)** The average integral optical density (IOD) of MACF1 positive cell on bone surface. ***Note:*** KD: MC3T3-E1 cells with stable lnc-PMIF knockdown, KD-NC: MC3T3-E1 cells with stable nonsense control RNA transfection, OE: MC3T3-E1 cells with stable lnc-PMIF overexpression, OE-NC: MC3T3-E1 cells with stable nonsense control RNA overexpression. For *in vivo* assay, n=3~4 mice per group. All data were expressed as mean ± SD. *$P < 0.05$ by Student's *t*-test.

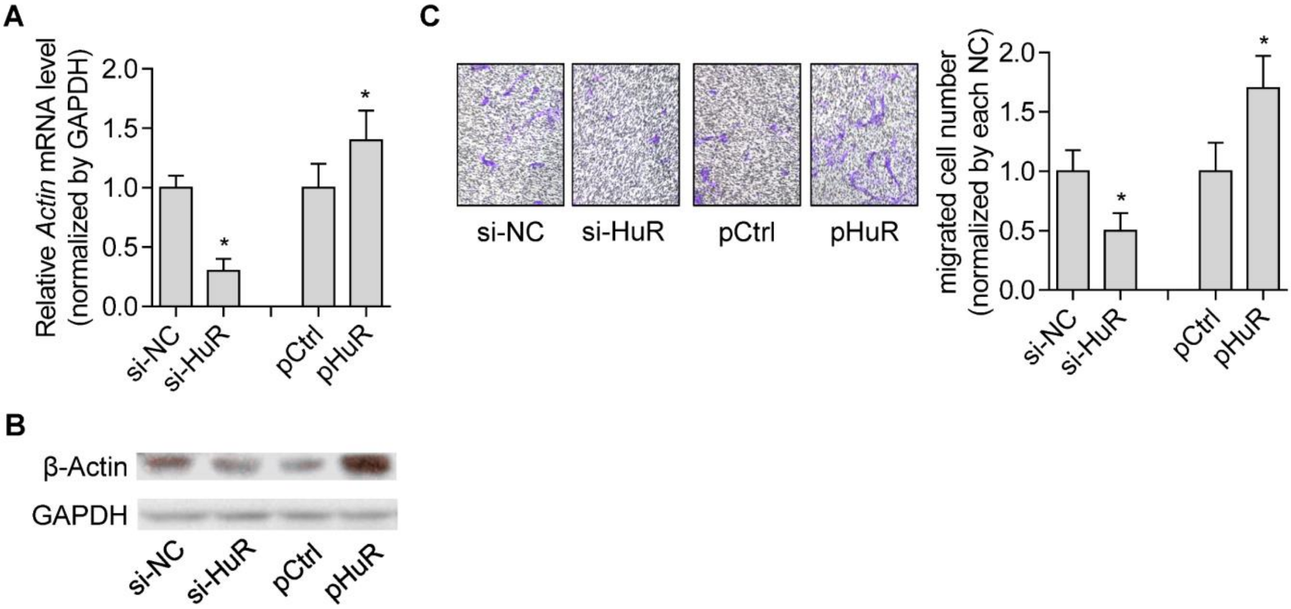

Figure S7 HuR regulate β-actin to promote OPC migration *in vitro*. (A) QPCR analysis of the β-actin mRNA expression in MC3T3-E1 cells transfected with HuR siRNA, NC-siRNA, HuR-expressing plasmid or control plasmid respectively. **(B)** Western blot analysis of β-actin and GAPDH protein expression in MC3T3-E1 cells transfected with HuR siRNA, NC-siRNA, HuR-expressing plasmid or control plasmid respectively. **(C)** Transwell migration assay on MC3T3-E1 cells transfected with HuR siRNA, NC-siRNA, HuR-expressing plasmid or control plasmid respectively. Left: representative images of the migrated cells. Right: the number of migrated cells. **(D)** Wound healing assay on MC3T3-E1 cells transfected with HuR siRNA, NC-siRNA, HuR-expressing plasmid or control plasmid respectively. Left: representative images. Right: quantification analysis of migration distance. ***Note:*** All *in vitro* experiments were conducted in triplicates. All data were expressed as mean ± SD. ns: not statistically significant, $^*P < 0.05$, $^{**}P < 0.01$ by Student's *t*-test.

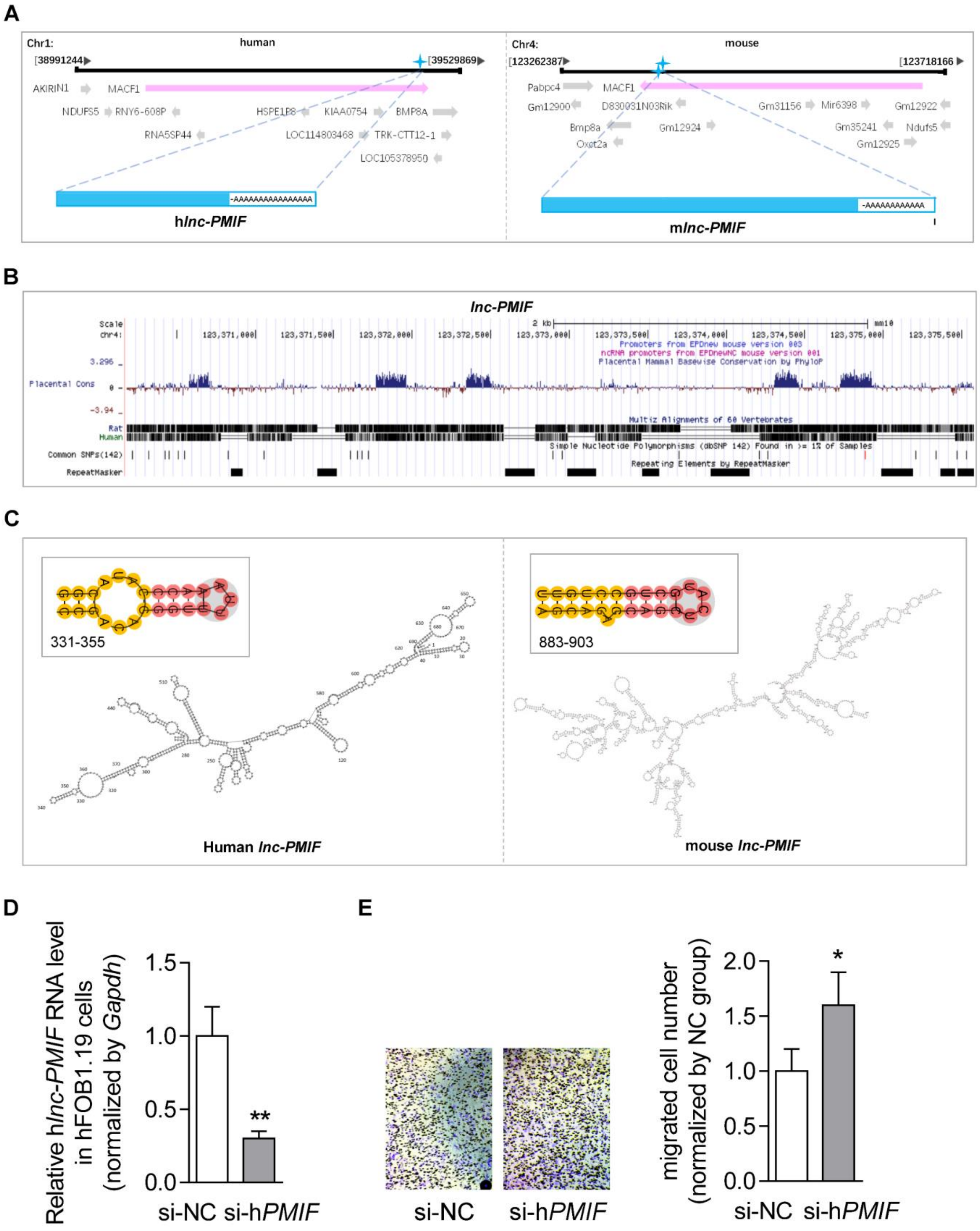
A
Chr1:
human
[38991244
[39529869
AKIRIN1
MACF1
NDUFS5
RNY6-608P
HSPE1P8
KIAA0754
BMP8A
RNA5SP44
LOC114803468
TRK-CTT12-1
LOC105378950
-AAAAAAAAAAAAAAA
hlnc-PMIF
Chr4:
mouse
[123262387
[123718166
Pabpc4
MACF1
Gm12900
D830031N03Rik
Gm31156
Mir6398
Gm12922
Bmp8a
Gm12924
Gm35241
Ndufs5
Oxct2a
Gm12925
-AAAAAAAAAAAA
mlnc-PMIF
B
lnc-PMIF
Scale
chr4:
2 kb
mm10
123,371,000
123,371,500
123,372,000
123,372,500
123,373,000
123,373,500
123,374,000
123,374,500
123,375,000
123,375,500
Promoters from EPDnew mouse version 003
ncRNA promoters from EPDnewNC mouse version 001
Placental Mammal Basewise Conservation by PhyloP
3.296
Placental Cons
0
-3.94
Multiz Alignments of 60 Vertebrates
Rat
Human
Simple Nucleotide Polymorphisms (dbSNP 142) Found in >= 1% of Samples
Common SNPs(142)
Repeating Elements by RepeatMasker
RepeatMasker
C
331-355
883-903
Human lnc-PMIF
mouse lnc-PMIF
D
Relative hlnc-PMIF RNA level in hFOB1.19 cells (normalized by Gapdh)
1.5
1.0
0.5
0.0
**
si-NC
si-hPMIF
E
si-NC
si-hPMIF
migrated cell number (normalized by NC group)
2.0
1.5
1.0
0.5
0.0
*
si-NC
si-hPMIF

Figure S8 The human Lnc-PMIF ortholog suppresses human OPC migration. (A) Schematic diagram of Macf1 and lnc-PMIF gene loci in human (left) and mouse (right). **(B)** The base-wise conservation of human/mouse lnc-PMIF gene at the corresponding gene locus (by UCSC, http://genome.ucsc.edu/). **(C)** The predicted secondary structure of human lnc-PMIF (left), mouse lnc-PMIF (right) (by RNAfold, http://rna.tbi.univie.ac.at//cgi-bin/RNAWebSuite/RNAfold.cgi) and their predicted motif binding to HuR (box) (by catRAPID, http://service.tartaglialab.com/page/catrapid_group). The red color indicates strong confidence for the prediction of each base. **(D)** QPCR analysis of has lnc-PMIF expression in human osteoblast precusor hFOB 1.19 cells transfected with has lnc-PMIF siRNA or NC siRNA. **(E)** Transwell migration assay on hFOB 1.19 cells transfected with has lnc-PMIF siRNA or NC siRNA. ***Note:*** All *in vitro* experiments were conducted in triplicates. All data were expressed as mean ± SD. ns: not statistically significant, *$P < 0.05$, **$P < 0.01$ by Student's *t*-test.

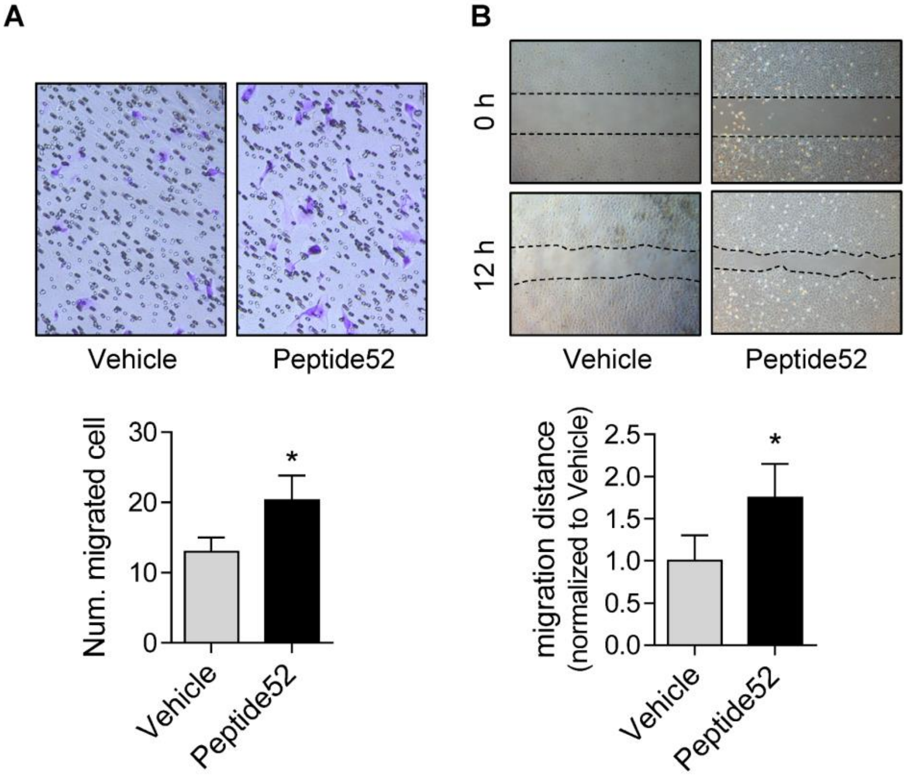

Figure S9 Cell migration assay of hFOB1.19 cells with or without exogenous supplementation of Peptide52. (A) Wound healing assay. Left: Representative images of the migrated cells. Right: the migration distance. **(B)** Transwell migration assay. ***Note:*** n = 3. *P < 0.05 by Student's *t*-test.

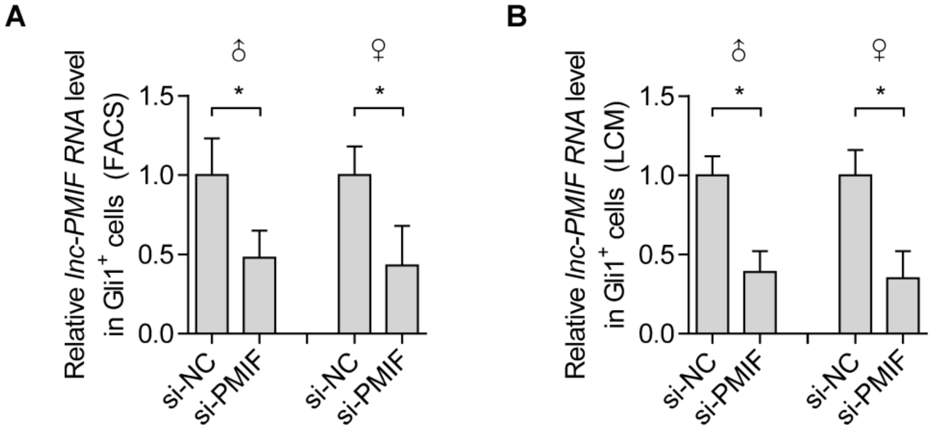

Figure S10 The lnc-PMIF knockdown efficiency in Gli1+ OPCs. (A) QPCR analysis of lnc-PMIF in the Gli1+ OPCs isolated from the cancellous bone region by fluorescent activated cell sorting (FACS) in different groups. **(B)** QPCR analysis of lnc-PMIF in the Gli1+ OPCs collected around the calcein labeled bone formation surface by laser capture microdissection (LCM) in different groups. ***Note:*** n=3~5 mice per group. All data were expressed as mean ± SD. *P < 0.05, **P < 0.01 by Student's *t*-test.

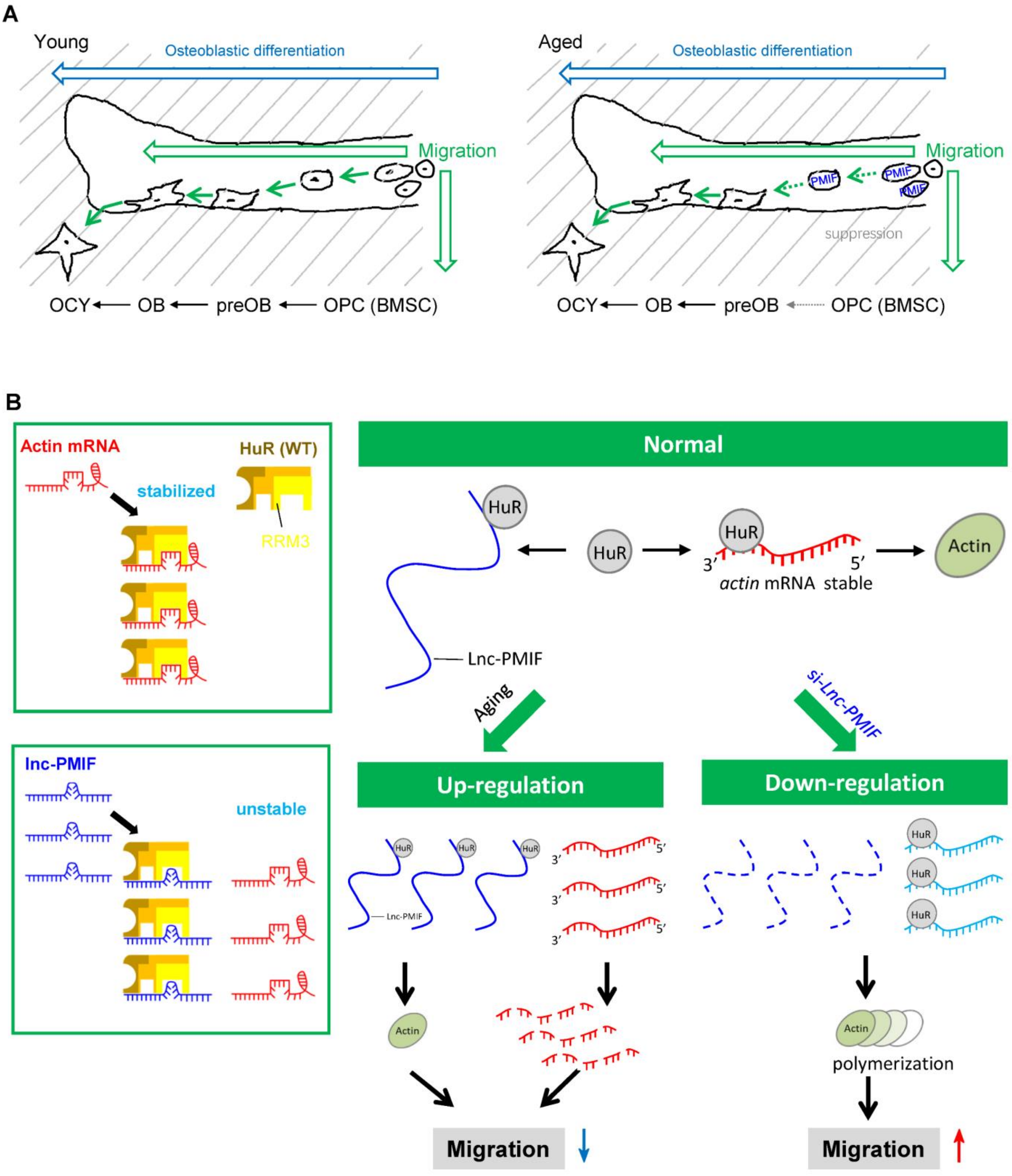

Figure S11 The proposed lnc-PMIF-mediated regulatory mechanism on OPC migration. (A) Schematic diagram showing that the aberrantly upregulated lnc-PMIF suppresses aged OPCs migrating to bone formation surface. **(B)** Schematic diagram showing that lnc-PMIF bind to the RRM3 of HuR for interrupting the HuR-β-actin mRNA interaction to inhibit β-actin expression for suppressing OPC migration.

Table S1. List and sequence of siRNAs, primers and probes utilized for experiments

Primer name	Sequences (5' to 3')
human Lnc-PMIF Primer-F	GGGATAGGCTCGTTGGTGAC
human Lnc-PMIF Primer-R	TCGGAGCTGAAGAACAGCAG
human Gapdh Primer-F	CCTCTGACTTCAACAGCGAC
human Gapdh Primer-R	TCCTCTTGTGCTCTTGCTGG
mus ACTB Primer-F	CCTGTGCTGCTCACCGAGG
mus ACTB Primer-R	TGAAGCTGTAGCCACGCTCG
mus Alp Primer-F	GTTGCCAAGCTGGGAAGAACAC
mus Alp Primer-R	CCCACCCCGCTATTCCAAAC
mus Bmp8a Primer-F	TGCCTATTACTGTGAGGGGGA
mus Bmp8a Primer-R	AGCAGGCTACTGTGGTACTGA
mus CollαI Primer-F	GAAGGCAACAGTCGATTCACC
mus CollαI Primer-R	GACTGTCTTGCCCCAAGTTCC
mus D830031N03Rik Primer-F	ATGCTGTAACACCGATGCCA
mus D830031N03Rik Primer-R	TCCTCTGGGATGGGCACTAT
mus Dynll1 Primer-F	TTTGTCCCTGCCAAGTACTG
mus Dynll1 Primer-R	CTTAACTGCCCTATCTGTGGTC
mus Gapdh Primer-F	TGCACCACCAACTGCTTAG
mus Gapdh Primer-R	GGATGCAGGGATGATGTTC
mus HuR Primer-F	ATGCTGCTGAACAGACTTCG
mus HuR Primer-R	TGTCTAATGGTTATGAAGACCACA
mus Macf1 Primer-F	GAAAACATTCACCAAGTGGGTCAAC
mus Macf1 Primer-F	TGTCCATCCCGAAGGTCTTCATAG
mus Mmp2 Primer-F	CCTTCACTTTCC TGGGCAACA
mus Mmp2 Primer-R	ATGGCATGGCCGAACTCAT
mus Ndufs5 Primer-F	GGGCGAAAAAGGAGTGCAAG
mus Ndufs5 Primer-R	AGGTGGAGGGGTGTATTTGC
mus Ocn Primer-F	GAACAGACTCCGGCGCTA
mus Ocn Primer-R	AGGGAGGATCAAGTCCCG
mus Oxct2a Primer-F	CGTGGGGTATCTGCTCTCCG
mus Oxct2a Primer-R	GACGGTAGACCCGTCCTTG
mus Pabpc4 Primer-F	ACCTGGCTGGGAAAATCACC
mus Pabpc4 Primer-F	AGAGGTAGCAGCAGCAACAG
mus Runx2 Primer-F	CGCCCCTCCCTGAACTCT
mus Runx2 Primer-R	GCCTGCCTGGGATCTGTA

Primer name	Sequences (5’ to 3’)
mus Lnc-PMIF Primer-F1	TTCCTGGTAACCCTGTAAC
mus Lnc-PMIF Primer-R1	GCTGATATAGCAAGTCAAGT
mus Lnc-PMIF siRNA-1	CCUUAGGUGCCUUUAGAAAdTdT
mus Lnc-PMIF siRNA-2	UUUCUAAAGGCACCUAAGGdTdT
Scramble si -mus Lnc-PMIF -1	AGCGUAUCAAUCGUUACUGTT
Scramble si -mus Lnc-PMIF -2	CAGUAACGAUUGAUACGCUTT

Table S2. Primer for lnc-PMIF qPCR and RACE

Usage	Primer Name	Sequences (5' to 3')
qPCR (Taqman Probe)	Lnc-PMIF-1-F	GATGCTGGGAATGGTTCAGTATAT (Forward)
	Lnc-PMIF-1-R	TGCACAAACACACCAGAGGA (Reverse)
	Lnc-PMIF-Probe	TGCCACCAAGCCTGACAACCTGAG (Probe)
3'RACE	Lnc-PMIF-1 Outer	CAGGTGTGGCTGTTAGCCCT
	3'RACE Outer	TACCGTCGTTCCACTAGTGATTT
	Lnc-PMIF-1 Inner	TGATGCCTTGGACAGACT
	3'RACE Inner	CGCGGATCTTCCACTAGTGATTTCACTATAGG
5'RACE	5'RACE Outer	CATGGCTACATGCTGACAGCTA
	Lnc-PMIF-1 Outer	AGCCACTCCAACAGCATA
	5'RACE Inner	CGCGGATCCACAGCCTACTGATGATCAGTCGATG
	5'RACE Inner	GATTGCGAAGGAAGAACC

Table S3. A List of lncPMIF-binding protein detected by Mass Spectra.

No.	ID	Description
1	P62242	40S ribosomal protein S8
2	Q8BGJ5	Q8BGJ5_Mouse MCG13402, isoform CRA_a
3	Q9CR57	60S ribosomal protein L14
4	P84099	60S ribosomal protein L19
5	Q99020	Heterogeneous nuclear ribonucleoprotein A/B
6	Q9CQF3	Cleavage and polyadenylation specificity factor subunit 5
7	P49312	Heterogeneous nuclear ribonucleoprotein A1
8	Q9CQE8	UPF0568 protein C14orf166 homolog
9	P27048	Small nuclear ribonucleoprotein-associated protein B
10	Q9CQI7	U2 small nuclear ribonucleoprotein B''
11	Q62348	Translin
12	P62908	40S ribosomal protein S3
13	P43277	Histone H1.3
14	Q9CX86	Heterogeneous nuclear ribonucleoprotein A0
15	P62960	Nuclease-sensitive element-binding protein 1
16	Q60668	Heterogeneous nuclear ribonucleoprotein D0
17	Q8VEK3	Heterogeneous nuclear ribonucleoprotein U
18	P97351	40S ribosomal protein S3a
19	P70372	ELAV-like protein 1 (HuR) ☆
20	P07724	Serum albumin

Additional Data File 1. Mice LncPMIF sequence.

CAGGTGTGGCTGTTAGCCCTGGAGCGTCAGCGGAAGCTGAATGATGCCTTGGACAGACT
GGAGGAGTTGAAAGAATTTGCCAACTTTGACTTTGATGTCTGGAGGAAAAAGTATATGCG
TTGGATGAATCATAAAAAATCTCGAGTCATGGATTTCTTCCGGCGTATTGACAAGGACCAG
GATGGGAAGATAACACGTCAGGAGTTTATCGATGGCATTTTAGCATCTAAGTTCCCAACCA
CCAAGTTAGAGATGACAGCTGTGGCCGACATTTTTGACAGAGATGGGGATGGCTACATTG
ACTACTATGAATTTGTGGCTGCCTTACATCCCAACAAGGATGCCTATCGGCCAACAACTGA
TGCAGATAAGATTGAAGATGAGGTCACAAGACAAGTGGCTCAGTGCAAGTGTGCAAAAA
GATTCCAAGTGGAACAGATTGGAGAGAATAAATACCGGGTAAGGAAGAGAAAAGCCAACC
ATTTGTGGAGGTCATTGCCTCCGGGGTCATCCTGACACACAGCAGAGCAGCTCATGCTG
CCTGTTCCCTCCTGCTGCCTCCAGAGGCCCAGAACCCAGGCCTTCATCCCTAGGTGTCA
AGTTTCATGCCCTGTGTAGTTCCATCTGAAAAGCTACCATTATTACCCAGGTAGACTGTAG
CTTATAGTTGACATGGTAGAAAATATTTCATAGCCCTCATCTCACTGCTGAGCAAAACCTTA
GGTGCCTTTAGAAACAGTTTTATTAAAGGACTTGTGAAGCAGAGTCTGAGATTCTTCCTGT
AATCCCAGAACTTGGGAGGCAAAGGCAGAAGCAGAAGCACCATTGCAAGTTCAAGACCA
ACTCTACATAGTGAGTCGCAGCTAACCAAGGCCATATCACAAAAATGTCTCAGATATTTTTA
AAAAGCATGGATCAGGATGCTGGGAATGGTTCAGTATATAAAGGTGCTTGCCACCAAGCC
TGACAACCTGAGTTCAAGTCCTAAAGGGGAAACACGCACCCCAAAATTGTCCTCTGGTGT
GTTTGTGCACATATACATTTTCTTTTTTGTTGTTGTTGTTTTGTTTTTTGGGCTTTTTGTTCT
GTTTTAATTTTTTTCCATTAAAAAAAAAGAGTGATGGTTCATCCACAAGAACTGGTCATTGT
GAGCCTACTGGCTCCCATGCTAATAGAAGACGATGTGTGAAAGCCCCCAACACATAGCTA
GTGTTGCTGTGCTGAGTTAGGCTACAACTGGAACCTACATGCCCTGGAGAGCCGGGCTC
AGTGATCTTCCCTGGAAAGACTTGTTGGGTATAATCAGAAGTAACAGGAAACTATGCTTAA
ATGTTAGCATCTCTTAGGACTAGAGTCAGGGATGTAGTAGAGACATAAACCAGGACTCCTA
TATGCATCAGCTTGTCAAGGGACCCTGAAATTTTAAGGAAAAAAAAAAACAACAAAAAAAA
AAAA

Additional Data File 2. Human Lnc-PMIF sequence.

TGCTACTGCAAGCAATTTAATACAAAAGTGTTCTTGTTTCTAAACAGTTCATTGGTAGAGCT
TCAGTTTCTGCCTCAATTAAAACCAATTGAGATAATCACACAGCAACATGGTCGGAGCTGA
AGAACAGCAGNTAAAAAACAGGAGCCCCAGACAGCCCCTTTTCATGGTCACTTGGTCAG
TTGACTTTATTACGCACAAAAAAAGACGAAAGCAAACAGGACTGGTTAACAAGTGTGCTG
GTGAACTCCATTAAAGGACGAGCACGTGTTTGTCTTACGTCCACATTTTCAGAACAGGCT
CGTGGAATTCATTCCTCAGTTTGTGGGAAGTTGATCGACCATTTAAATAAATAGCAAAAAA
AGTGGATAAAACAAATCTGAAATTCACAATGTCACCAACGAGCCTATCCCTTGCCAAGGGT
CTGGGTCCTGGTGAACACAAGAGTTACTGGTGTCTAGGCTGGATAGGGAGCAACAAATC
AGTTTAAAACCCCAAATGTTCTGACGTTTTCTGAACACTTACATTTACCCCTGAATTCTAGC
AGCTATTTGTCCTTTAAACACCCAGTACTGAAACAATATCCAAAGACAGTATTGCAATGAAC
TCCTTTTGCTGTATCATGTGCAAATGTTACTTAGATGAGGTTCCCTCTCTTTAGCTAAAAAT
CACAAAAAAAAAAAAAAAAAA

Additional Data File 3. The sequence of HuR-RRM3-Peptide52. (highlighted in yellow)

> mice-HuR (NP_034615.2 ELAV-like protein 1)

MSNGYEDHMAEDCRDDIGRTNLIVNYLPQNMTQEELRSLFSSIGEVESAKLIRDKVAGHSLGYGFVNYVTAKDAERAISTLNGLRLQSKTIKVSYARPSSEVIKDANLYISGLPRTMTQKDVEDMFSRFGRIINSRVLVDQTTGLSRGVAFIRFDKRSEAEEAITSFNGHKPPGSSEPITVKFAANPNQNKNMALLSQLYHSPARRFGGPVHHQAQRFRFSPMGVDHMSGISGVNVPGNASSGWCIFIYNLGQDADEGILWQMFGPFGAVTNVKVIRDFNTNKCKGFGFVTMTNYEEAAMAIASLNGYRLGDKILQVSFKTNKSHK

>human-HuR (NP_001410.2 ELAV-like protein 1)

MSNGYEDHMAEDCRGDIGRTNLIVNYLPQNMTQDELRSLFSSIGEVESAKLIRDKVAGHSLGYGFVNYVTAKDAERAINTLNGLRLQSKTIKVSYARPSSEVIKDANLYISGLPRTMTQKDVEDMFSRFGRIINSRVLVDQTTGLSRGVAFIRFDKRSEAEEAITSFNGHKPPGSSEPITVKFAANPNQNKNVALLSQLYHSPARRFGGPVHHQAQRFRFSPMGVDHMSGLSGVNVPGNASSGWCIFIYNLGQDADEGILWQMFGPFGAVTNVKVIRDFNTNKCKGFGFVTMTNYEEAAMAIASLNGYRLGDKILQVSFKTNKSHK
